# Difference in carcinogenicities of two different vapor grown carbon fibers with different physicochemical characteristics induced by intratracheal instillation in rats

**DOI:** 10.1186/s12989-023-00547-5

**Published:** 2023-09-28

**Authors:** Kei Sato, Hiroko Fukui, Yuji Hagiwara, Ryoji Ogawa, Ayako Nishioka, Takamasa Numano, Taiki Sugiyama, Mayumi Kawabe, Yukinori Mera, Tadashi Yoneda

**Affiliations:** 1 Chemical Management Department, Resonac Corporation, Tokyo Shiodome Bldg.,1-9-1, Higashi-Shimbashi, Minato-ku, Tokyo 105-7325 Japan; 2 DIMS Institute of Medical Science, Inc., 64 Goura, Nishiazai, Azai-cho, Ichinomiya-shi, Aichi 491-0113 Japan

**Keywords:** Vapor grown carbon fiber, Multi-walled carbon nanotube, Intratracheal instillation, 2-Year carcinogenicity study, Rat, Carbon fiber, Mesothelioma

## Abstract

**Background:**

Carbon fibers are high aspect ratio structures with diameters on the submicron scale. Vapor grown carbon fibers are contained within multi-walled carbon tubes, with VGCF™-H commonly applied as a conductive additive in lithium-ion batteries. However, several multi-walled carbon fibers, including MWNT-7, have been reported to induce lung carcinogenicity in rats. This study investigated the carcinogenic potential of VGCF™-H fibers in F344 rats of both sexes with the vapor grown carbon fibers VGCF™-H and MWNT-7 over 2 years. The carbon fibers were administered to rats by intratracheal instillation at doses of 0, 0.016, 0.08, and 0.4 mg/kg (total doses of 0, 0.128, 0.64, and 3.2 mg/kg) once per week for eight weeks and the rats were observed for up to 2 years after the first instillation.

**Results:**

Histopathological examination showed the induction of malignant mesothelioma on the pleural cavity with dose-dependent increases observed at 0, 0.128, 0.64, and 3.2 mg/kg in rats of both sexes that were exposed to MWNT-7. On the other hand, only two cases of pleural malignant mesothelioma were observed in the VGCF™-H groups; both rats that received 3.2 mg/kg in male. The animals in the MWNT-7 groups either died or became moribund earlier than those in the VGCF™-H groups, which is thought related to the development of malignant mesothelioma. The survival rates were higher in the VGCF™-H group, and more carbon fibers were observed in the pleural lavage fluid (PLF) of the MWNT-7 groups. These results suggest that malignant mesothelioma is related to the transfer of carbon fibers into the pleural cavity.

**Conclusions:**

The intratracheal instillation of MWNT-7 clearly led to carcinogenicity in both male and female rats at all doses. The equivocal evidence for carcinogenic potential that was observed in male rats exposed to VGCF™-H was not seen in the females. The differences in the carcinogenicities of the two types of carbon fibers are thought due to differences in the number of carbon fibers reaching the pleural cavity. The results indicate that the carcinogenic activity of VGCF™-H is lower than that of MWNT-7.

**Supplementary Information:**

The online version contains supplementary material available at 10.1186/s12989-023-00547-5.

## Background

Carbon fibers with nanoscale to submicron-scale diameters and high aspect ratios have excellent physicochemical and electrical properties. Multi-walled carbon nanotubes (MWCNTs) are carbon fibers with particularly excellent properties that have been widely applied widely in industry and several are commercially available. The production of MWCNTs has increased in recent years; however, the needle-like/fibrous structure and high aspect ratio of MWCNTs is similar to asbestos and there is concern that these fibrous materials may be bio-persistent [1, 2], resulting in pulmonary toxicity. MWNT-7, which has a needle-like/fibrous structure and a high aspect ratio, has been found to promote the development of lung carcinogenesis in mice treated with whole-body inhalation [3]. Moreover, in a carcinogenicity study using rats, MWNT-7 induced lung carcinoma not only in animals treated with whole-body inhalation [4], but instigated the development of pleural malignant mesothelioma in animals to whom fibers were delivered via intratracheal instillation [5]. The IARC has classified MWNT-7 as group 2B (possibly carcinogenic to humans) [6] based on the report of an inhalation carcinogenicity study that used a carcinogenic initiator [3] and the increased incidence of mesothelioma in the abdominal cavity of rats that were subjected to intraperitoneal [7] and intrascrotal administration [8] or p53 heterozygous mice treated with intraperitoneal injection [9]. These reports indicate that materials with needle-like/fibrous structures may be carcinogenic, rendering it necessary to obtain further information on the carcinogenicity of these materials for an accurate hazard assessment with respect to the carcinogenic potential in humans.

Vapor grown carbon fibers are formulated to enhance the electrical and thermal properties of high-performance materials with multi-walled carbon tube structures. VGCF™-H and MWNT-7, the test materials in this study, are vapor grown carbon fibers. The physicochemical properties of VGCF™-H, such as the diameter, length, and electrical properties differ considerably from those of MWNT-7 because of the different manufacturing conditions, and it has superior conductivity and dispersion. However, both are fibrous structures with a high aspect ratio in a broad sense. These factors are concerning, and it is unknown whether the continuous inhalation of VGCF™-H fibers may induce persistent lung toxicity and carcinogenicity. Delorme et al., reported that the inhalation of 0.54, 2.5, and 25 mg/m^3^ VGCF™-H over a 90-day period in rats led to a concentration-related small, but detectable, accumulation of extrapulmonary fibers in various organs and tissues over the whole-body. No adverse effects were observed in the tissues, and a no-observed-adverse-effect level of VGCF™-H for male and female rats of 0.54 mg/m^3^ was obtained [10]. However, MWNT-7 induced lung toxicity such as inflammation, indicated by bronchioalveolar lavage fluid (BALF) from whole-body inhalation of even 0.2 mg/m^3^, was observed in a 13-week study [11]. These reports suggest that the sub-chronic lung toxicity level of VGCF™-H is lower than that of MWNT-7. However, no studies have reported on the lung carcinogenicity of VGCF™-H, and its potential carcinogenicity remains unknown, and no studies have as yet compared the pulmonary carcinogenicity of VGCF™-H with that of MWNT-7.

Whole-body inhalation studies are usually performed over 2 years to evaluate lung carcinogenicity; however, reports on this type of experiment are limited because of the extensive facilities required for whole-body inhalation. However, the intratracheal instillation method is often used to evaluate the acute to sub-chronic lung toxicity of carbon fibers. We previously performed a 13-week toxicity study of VGCF™-H on rat lungs with MWNT-7 as a comparative material to obtain the dosage for a carcinogenicity study using intratracheal instillation [12]. The sub-chronic lung toxicity of VGCF™-H was evaluated by means of eight treatments with 0.2, 0.4, and 0.8 mg/kg (total doses of 1.6, 3.2, and 6.4 mg/kg, respectively) that were administered once per week and compared with 3.2 and 6.4 mg/kg of MWNT-7. Both BALF and histopathological analyses showed that the sub-chronic lung toxicity was dose-dependent increases for both MWNT-7 and VGCF™-H; however, the lung toxicity of VGCF™-H was expectedly lower than that of MWNT-7. A comparative study of the sub-chronic lung toxicity of VGCF™-H and MWNT-7 using both whole-body inhalation and intratracheal instillation produced comparable results indicating that the adverse effects of MWNT-7 as compared to VGCF™-H. Although some sub-chronic toxicity inhalation studies investigating carbon fibers have been reported, only limited studies evaluating carcinogenicity have been conducted owing to the difficulty of conducting whole-body inhalation. In addition, although MWNT-7 has been examined in most carcinogenicity studies, few reports of the chronic toxicity of other carbon fibers have been made. One carcinogenicity study that used carbon fibers other than MWNT-7 by Suzui et al. reported that the intratracheal instillation of MWCNT (NIKKISO) into rat lungs induced malignant mesothelioma and lung tumors [13]. Furthermore, Numano et al. reported that the intratracheal instillation of MWNT-7 into rats induced the transfer of carbon fibers from lungs to the thoracic cavity, causing pleural malignant mesothelioma in 95% of the studied animals [5]. On the other hand, it was reported that no malignant mesothelioma was observed in whole-body inhalation study of MWNT-7 [4]. It is unclear whether other vapor grown carbon fibers, including VGCF™-H, also differ in the development of malignant mesothelioma in whole-body inhalation and intratracheal studies. However, at least the sub-chronic lung toxicity observed for MWNT-7 and VGCF™-H was consistent in that MWNT-7 was more adverse in both whole-body inhalation and intratracheal instillation studies. Based on these results, it was considered that the intratracheal instillation method could be used to lung hazard identification and screening purpose. We therefore decided to investigate the lung carcinogenicity of VGCF™-H using intratracheal instillation. This paper describes the first chronic lung toxicity study of VGCF™-H in rats.

## Results

### Characteristics of carbon fibers

#### The primary particle morphology, dispersion state, and iron content of carbon fibers

To determine the differences in the physicochemical properties of VGCF™-H and MWNT-7, an investigation of the primary particle morphology and the average hydrodynamic diameter of the carbon fibers was performed by scanning electron microscopy (SEM) and dynamic light scattering (DLS). The VGCF™-H fiber was a diameter of 148 ± 52 nm, a length of 5.2 ± 2.7 μm and a surface area of 15 m^2^/g. The MWNT-7 fiber was a diameter of 75 ± 20 nm, a length of 9.0 ± 6.1 μm and a surface area of 25 m^2^/g. Additional file 1: Fig. S1 shows the distribution of the carbon fiber length and diameter of MWNT-7 and VGCF™-H used in this study. Both VGCF™-H and MWNT-7 fibers were straight in shape. The average hydrodynamic diameters of MWNT-7 and VGCF™-H were 656 ± 34 nm and 783 ± 43 nm, 514 ± 40 nm and 589 ± 23 nm, and 561 ± 63 nm and 613 ± 29 nm in the 0.008 mg/mL (for 0.128 mg/kg), 0.04 mg/mL (for 0.64 mg/kg), and 0.2 mg/mL (for 3.2 mg/kg) doses, respectively. The average hydrodynamic diameters of the fibers did not change when passed through the microsprayer aerosolizer (data not shown). Iron contents of MWNT-7 and VGCF™-H fiber was 4200 ppm and 9.7 ppm, but no other elements were detected for VGCF™-H fibers (Additional file 1: Table S1).

#### Dispersion state of carbon fibers in saline solution containing 0.3% Kolliphor P188

**Fig. 1:**
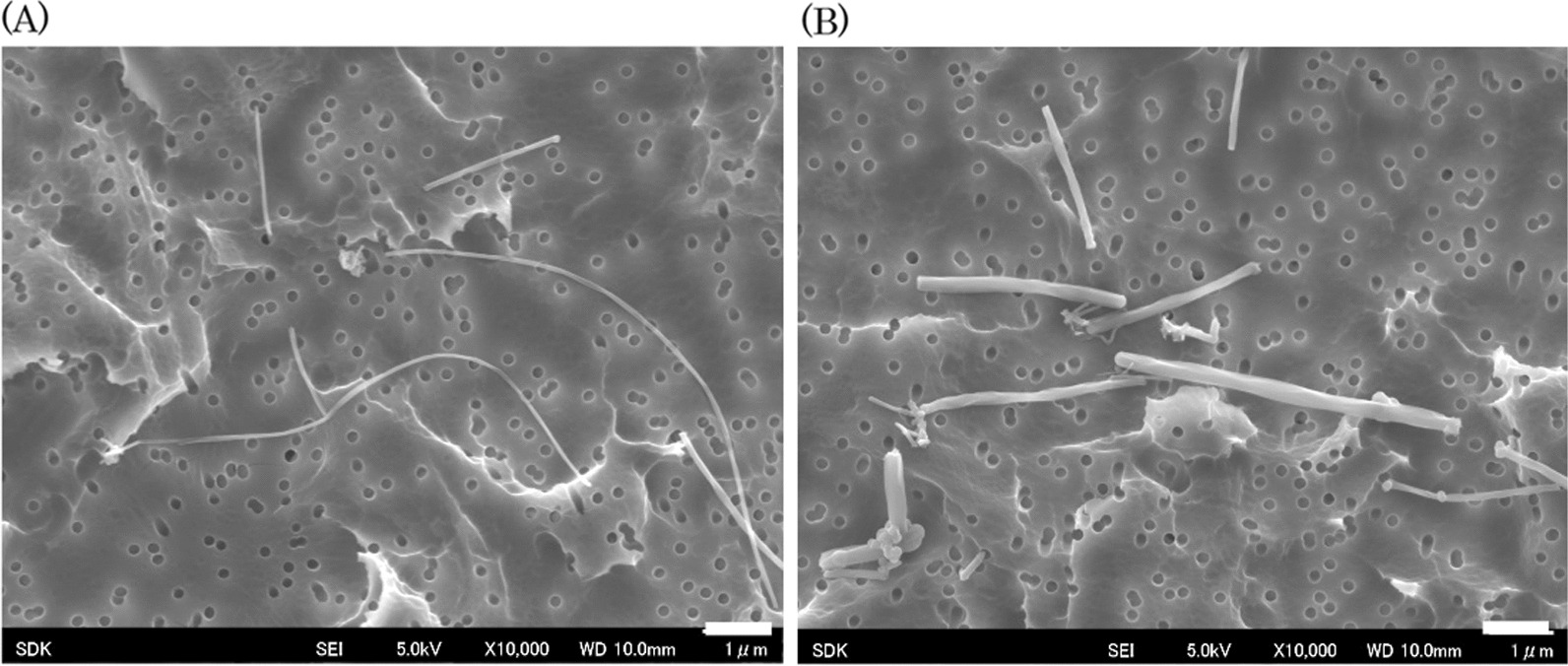
Representative scanning electron microscopic images of MWNT-7 (**A**) and VGCF™-H (**B**) fibers in the prepared solution. VGCF™-H fibers were shorter and thicker than MWNT-7 fibers. The scale bar indicates 1 μm

Figure 1 shows representative SEM images of carbon fibers in the prepared test material solution. SEM observation was performed to evaluate the dispersion state of the carbon fibers in the solution. VGCF™-H fibers were found to be shorter and thicker than MWNT-7 fibers, and both the MWNT-7 and VGCF™-H fibers dispersed as single straight fibers with no aggregation. These profiles were observed at all doses.

### Survival and body weight

**Fig. 2:**
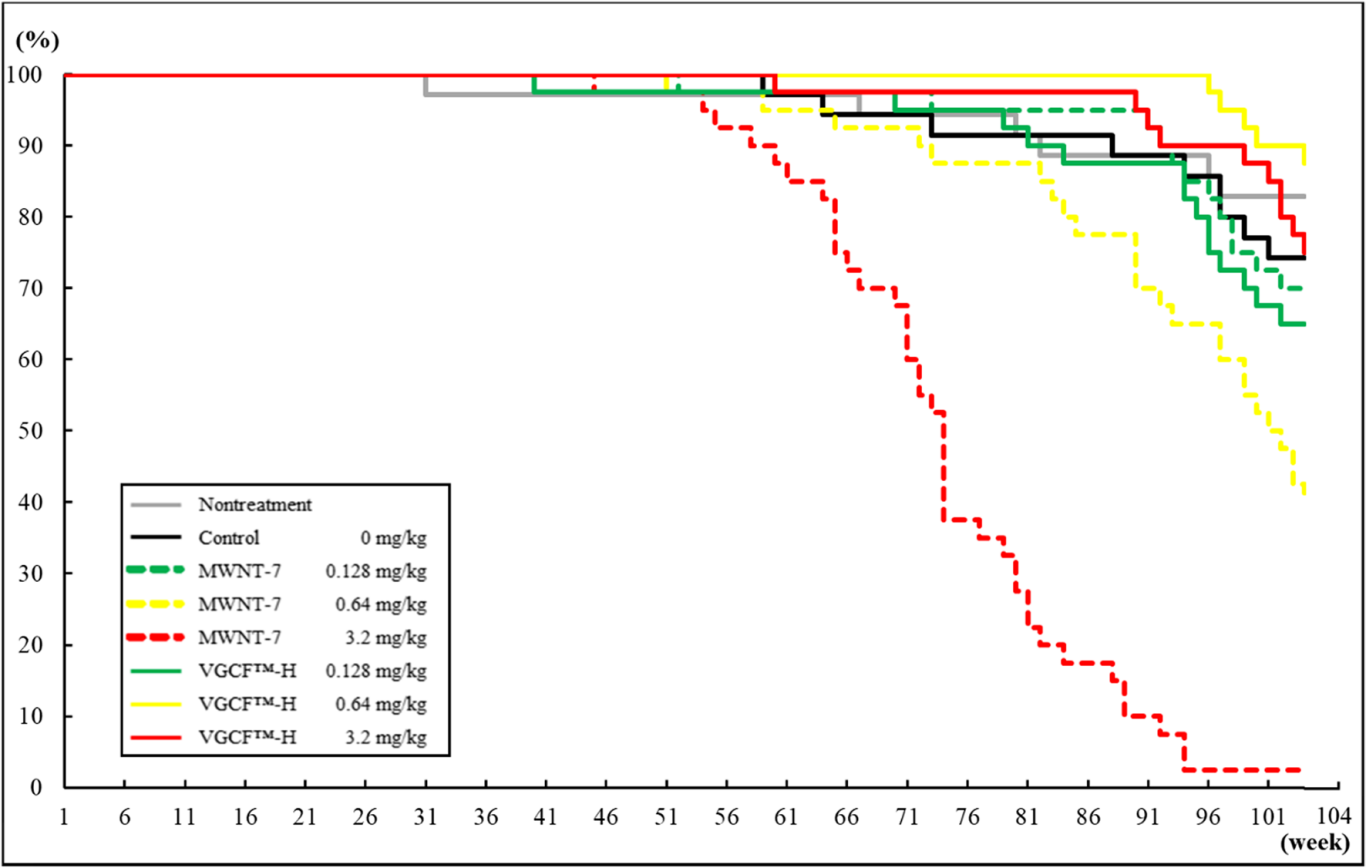
Kaplan–Meier survival plot for the male rats in the nontreatment, control, MWNT-7, and VGCF™-H groups in each dose

**Fig. 3:**
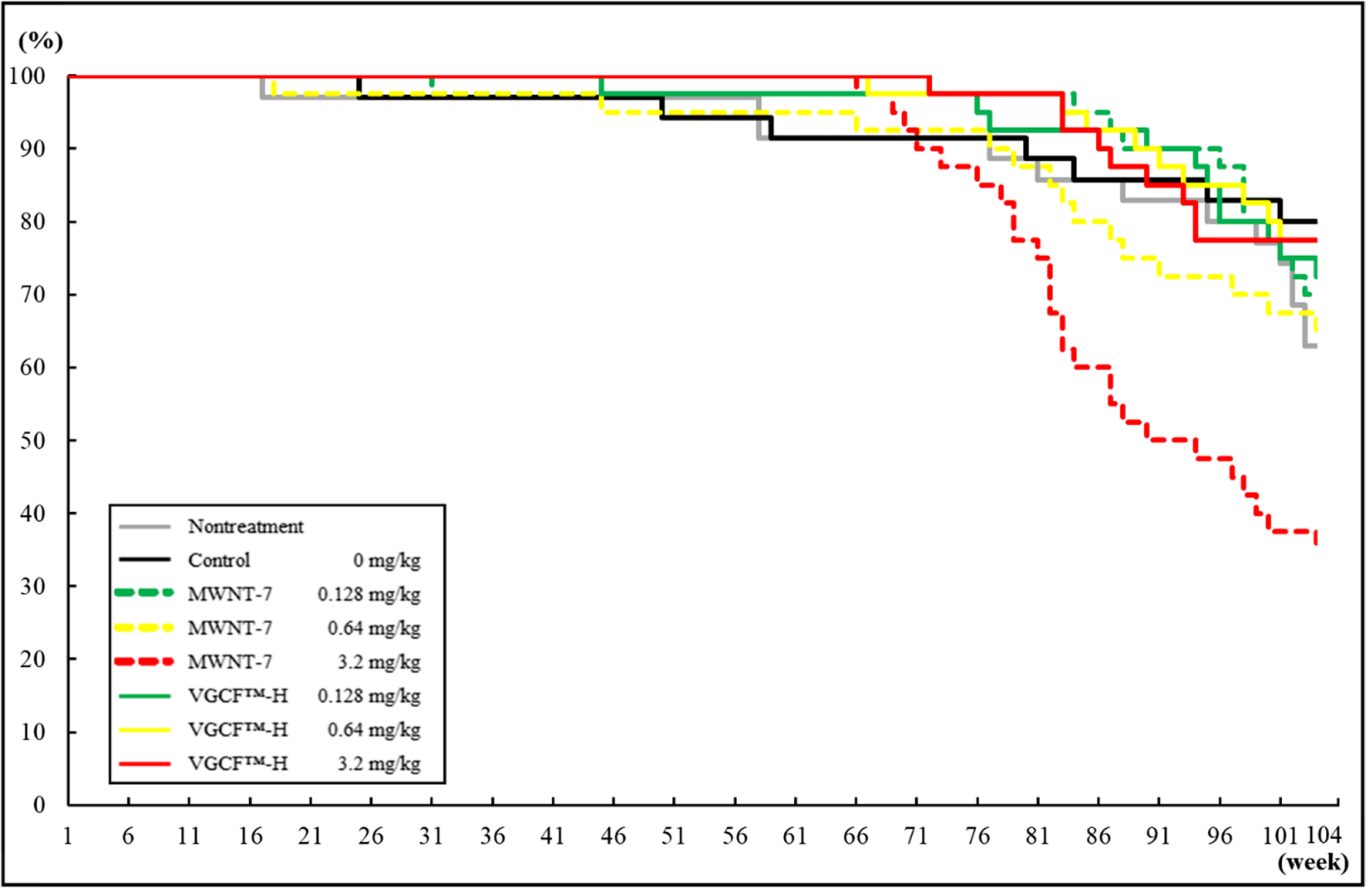
Kaplan–Meier survival plot for the female rats in the nontreatment, control, MWNT-7, and VGCF™-H groups in each dose

Figure 2 and 3 shows a Kaplan–Meier survival curve for male and female rats. Male rats in the 0.64 and 3.2 mg/kg MWNT-7 groups began to die 56 and 53 weeks into the experimental period, which was notably earlier than those in the VGCF™-H groups (Fig. 2). In a previous report describing a 2 year carcinogenicity study, the final survival rate of male and female F344 rats were 66.0–88.0% and 68.0–86.0%, respectively [14]. No significant change in the survival rates of male and female rats in the nontreatment and control groups (82.9% and 74.3%, and 62.9%, and 80.0%, respectively) was reported compared to the results obtained in previous studies, suggesting that the intratracheal instillation process had no effect on the survival rate. The final survival rates of male rats in the MWNT-7 groups were 70.0, 40.0, and 2.5%, and survival rates of 65.0, 87.5, and 75.0% observed in the VGCF™-H groups subjected to 0.128, 0.64, and 3.2 mg/kg, respectively. Importantly, the survival rates of male rats in the 0.64 and 3.2 mg/kg MWNT-7 groups were lower than those of all other groups. On the other hand, no clear decrease in the survival rate was observed in the male VGCF™-H groups, although the survival rate of the VGCF™-H group that received 0.128 mg/kg was slightly lower than that of the control group. Females in the 3.2 mg/kg MWNT-7 group began to die at week 65 in the experimental period (Fig. 3), which was earlier than the females in the other groups. The final survival rate at week-104 of the experimental period were 70.0, 65.0, and 35.0% for the female MWNT-7 groups, and 72.5, 77.5, and 77.5% for the female VGCF™-H groups at 0.128, 0.64, and 3.2 mg/kg, respectively. The survival rates of the 0.64 and 3.2 mg/kg MWNT-7 groups were therefore markedly lower than those of the VGCF™-H group. The survival rate of the all-female MWNT-7 groups was lower than that of the control group. The survival rate of the male MWNT-7 groups was lower than that of females, and no decreasing trends were observed in the survival rates of the male VGCF™-H groups at any dose, as were observed in the male MWNT-7 groups.

Body weights fluctuated slightly after instillation of MWNT-7 or VGCF™-H (data not shown), but no significant changes were observed in either group at week 104 of the experimental period (Table 1).

**Table 1:** Body weights at week 104 of the experimental period

Test article	Total dose (mg/kg)	Male (g)		Female (g)	
Nontreatment	-	412.0 ± 35.7	(29)	261.9 ± 36.8	(22)
Control	0	421.6 ± 47.3	(26)	272.6 ± 16.2	(28)
MWNT-7	0.128	412.5 ± 42.1	(28)	272.9 ± 36.0	(28)
MWNT-7	0.64	417.8 ± 39.3	(16)	258.1 ± 32.3	(26)
MWNT-7	3.2	405.4	(1)	269.1 ± 17.3	(15)
VGCF™-H	0.128	412.5 ± 64.6	(26)	264.1 ± 43.5	(30)
VGCF™-H	0.64	413.3 ± 46.3	(35)	270.5 ± 29.9	(31)
VGCF™-H	3.2	417.8 ± 44.9	(30)	274.0 ± 17.6	(31)

Number in parentheses indicates the number of animals examined

The values indicate as mean ± S.D

### Evaluation of inflammation and tissue damage by carbon fibers

#### Analysis of inflammatory cells in the PLF

Table 2 shows the cellular component of the PLF at week 13 of the experimental period. No significant changes were observed in the nontreatment and control groups, with both males and females unaffected in terms of the studied parameters. The number of white blood cells (WBC) and differential leukocytes increased significantly in the male MWNT-7 groups, except for basophils. No significant changes were observed in the PLF from males and females in the VGCF™-H groups, except for a significant increase in the numbers of lymphocytes and eosinophils in the male 3.2 mg/kg VGCF™-H group. A significant increase was observed in the WBC and differential leukocytes of the female MWNT-7 groups, except for neutrophils and basophils, while no significant increase was observed in either WBC or differential leukocytes in the female VGCF™-H groups at week 13 of the experimental period.

**Table 2:** Cellular components in the PLF at week 13 of the experimental period

Test article	Total dose (mg/kg)		Female					
			WBC (× 10^2^/µL)	Leukocytes, differential				
				Lymphocytes	Neutrophils	Eosinophils	Basophils	Monocytes
				(× 10^2^/µL)	(× 10^2^/µL)	(× 10^2^/µL)	(× 10^2^/µL)	(× 10^2^/µL)
Nontreatment	–	(5)	20.2 ± 5.6	1.4 ± 0.3	3.1 ± 1.0	1.3 ± 0.4	0.0 ± 0.0	14.4 ± 4.0
Control	0	(5)	20.3 ± 6.4	1.5 ± 0.4	3.2 ± 1.0	1.2 ± 0.4	0.0 ± 0.0	14.4 ± 4.9
MWNT-7	0.128	(5)	30.2 ± 3.3	2.3 ± 0.4^#^	2.4 ± 0.5	2.1 ± 0.4^#^	0.0 ± 0.0	23.4 ± 2.2^#^
MWNT-7	0.64	(5)	39.3 ± 9.9^#^	2.1 ± 0.5	2.8 ± 1.1	4.2 ± 1.5^#^	0.0 ± 0.0	30.2 ± 7.1^#^
MWNT-7	3.2	(5)	41.8 ± 14.8^##^	1.5 ± 0.4	3.4 ± 0.7	6.5 ± 2.5^#^	0.0 ± 0.0	30.4 ± 12.3
VGCF™-H	0.128	(5)	22.1 ± 4.7	1.9 ± 0.6	3.1 ± 0.6	1.3 ± 0.4	0.0 ± 0.0	15.9 ± 3.9
VGCF™-H	0.64	(5)	20.6 ± 5.4	1.7 ± 0.7	2.7 ± 1.3	1.3 ± 0.3	0.0 ± 0.0	14.9 ± 3.6
VGCF™-H	3.2	(5)	23.9 ± 6.7	1.9 ± 0.6	1.8 ± 0.6	1.4 ± 0.4	0.0 ± 0.0	18.7 ± 5.5

Test article	Total dose (mg/kg)		Male					
			WBC (× 10^2^/µL)	Leukocytes, differential				
				Lymphocytes	Neutrophils	Eosinophils	Basophils	Monocytes
				(× 10^2^/µL)	(× 10^2^/µL)	(× 10^2^/µL)	(× 10^2^/µL)	(× 10^2^/µL)
Nontreatment	–	(5)	35.6 ± 7.7	2.2 ± 0.9	3.4 ± 1.5	1.6 ± 0.2	0.0 ± 0.0	28.4 ± 5.7
Control	0	(5)	28.5 ± 10.5	1.7 ± 0.5	3.0 ± 0.9	1.3 ± 0.3	0.0 ± 0.0	22.6 ± 9.3
MWNT-7	0.128	(5)	37.6 ± 10.6	2.5 ± 0.6^#^	3.1 ± 0.7	2.6 ± 0.6^#^	0.0 ± 0.0	29.4 ± 9.2
MWNT-7	0.64	(5)	69.7 ± 10.2^##^	3.1 ± 0.6^##^	6.2 ± 1.8^##^	7.7 ± 1.7^#^	0.0 ± 0.0	52.8 ± 6.5^##^
MWNT-7	3.2	(5)	93.0 ± 12.1^##^	1.6 ± 0.2	6.3 ± 1.9^##^	14.9 ± 3.0^#^	0.0 ± 0.0	70.3 ± 9.0^##^
VGCF™-H	0.128	(5)	30.9 ± 3.0	1.9 ± 0.3	3.1 ± 0.7	1.3 ± 0.3	0.0 ± 0.0	24.6 ± 2.9
VGCF™-H	0.64	(5)	34.9 ± 8.0	2.4 ± 0.6	3.3 ± 1.2	1.4 ± 0.2	0.0 ± 0.0	27.7 ± 6.4
VGCF™-H	3.2	(5)	40.1 ± 7.8	2.6 ± 0.7^#^	3.1 ± 0.8	1.9 ± 0.4^#^	0.0 ± 0.0	32.5 ± 6.2

Number in parentheses indicates the number of animals examined. The values indicate as mean ± S.D

Significantly different from the Control group; ^#^*p* < 0.05, ^##^*p* < 0.01

Table 3 shows the cellular components of the PLF at week 104 of the experimental period. No significant increase was observed in the WBC or the differential leukocytes in all male VGCF™-H groups at week 104. Many of the male animals in the MWNT-7 group that received 3.2 mg/kg died by 104 weeks of the experiment, and the cellular components of the PLF could only be measured in one rat, with a significant decrease observed in the lymphocytes of the 0.64 mg/kg male MWNT-7 group thought to be the result of error as the changes were not dose dependent. There was a significant increase in the monocytes of the 3.2 mg/kg MWNT-7 group; however, no other significant changes were observed in any of the female groups.

**Table 3:** Cellular components in the PLF at week 104 of the experimental period

Test article	Total dose (mg/kg)		Male					
			WBC (× 10^2^/µL)	Leukocytes, differential				
				Lymphocytes	Neutrophils	Eosinophils	Basophils	Monocytes
				(× 10^2^/µL)	(× 10^2^/µL)	(× 10^2^/µL)	(× 10^2^/µL)	(× 10^2^/µL)
Nontreatment	–	(5)	46.6 ± 6.6	6.4 ± 2.0	1.4 ± 0.7	0.5 ± 0.6	0.0 ± 0.0	38.2 ± 6.3
Control	0	(5)	71.2 ± 48.0	11.6 ± 9.5	2.7 ± 1.6	1.9 ± 2.9	0.0 ± 0.0	54.9 ± 35.9
MWNT-7	0.128	(5)	55.1 ± 8.8	5.0 ± 1.1	2.2 ± 0.6	1.7 ± 0.7	0.0 ± 0.0	46.1 ± 7.8
MWNT-7	0.64	(5)	69.7 ± 7.5	3.2 ± 0.7^#^	2.0 ± 0.6	2.7 ± 1.2	0.0 ± 0.0	61.8 ± 6.7
MWNT-7	3.2	(1)	103.0	10.4	8.0	1.4	0.0	83.2
VGCF™-H	0.128	(5)	43.0 ± 14.5	9.8 ± 5.3	1.2 ± 0.4	0.2 ± 0.2	0.0 ± 0.0	31.8 ± 11.4
VGCF™-H	0.64	(5)	44.4 ± 5.0	4.8 ± 1.6	1.4 ± 0.4	0.5 ± 0.3	0.0 ± 0.0	37.7 ± 3.4
VGCF™-H	3.2	(5)	56.0 ± 5.7	6.7 ± 1.8	1.9 ± 0.3	1.1 ± 0.4	0.0 ± 0.0	46.2 ± 4.8

Test article	Total dose (mg/kg)		Female					
			WBC (× 10^2^/µL)	Leukocytes, differential				
				Lymphocytes	Neutrophils	Eosinophils	Basophils	Monocytes
				(× 10^2^/µL)	(× 10^2^/µL)	(× 10^2^/µL)	(× 10^2^/µL)	(× 10^2^/µL)
Nontreatment	–	(5)	28.9 ± 14.1	4.7 ± 3.3	0.7 ± 0.5	0.3 ± 0.2	0.0 ± 0.0	23.3 ± 10.5
Control	0	(5)	36.9 ± 13.1	7.0 ± 4.2	1.0 ± 0.3	0.4 ± 0.2	0.0 ± 0.0	28.6 ± 8.9
MWNT-7	0.128	(5)	34.4 ± 10.8	5.8 ± 6.1	0.7 ± 0.4	1.1 ± 0.7	0.0 ± 0.0	26.8 ± 8.5
MWNT-7	0.64	(5)	39.7 ± 8.8	4.4 ± 3.7	1.1 ± 1.0	1.6 ± 1.8	0.0 ± 0.0	32.6 ± 8.8
MWNT-7	3.2	(5)	61.9 ± 32.8	5.6 ± 6.8	1.3 ± 0.8	0.7 ± 0.4	0.0 ± 0.0	54.4 ± 25.7
VGCF™-H	0.128	(5)	29.1 ± 7.2	4.5 ± 2.9	0.6 ± 0.4	0.1 ± 0.2	0.0 ± 0.0	23.9 ± 4.2
VGCF™-H	0.64	(5)	31.8 ± 9.4	6.0 ± 5.8	0.6 ± 0.4	0.3 ± 0.3	0.0 ± 0.0	24.9 ± 5.3
VGCF™-H	3.2	(5)	42.7 ± 7.2	6.9 ± 3.4	0.7 ± 0.3	0.4 ± 0.2	0.0 ± 0.0	34.7 ± 4.1

Number in parentheses indicates the number of animals examined. The values indicate as mean ± S.D

Significantly different from the Control group; ^#^*p* < 0.05

A lower inflammatory response was observed in the VGCF™-H groups than the MWNT-7 groups. The data indicating the cellular component in the PLF at week 104 of the experimental period were less variable and the association between these results and carcinogenicity was unclear.

#### Analysis of clinical chemistry in the PLF

Table 4 shows the clinical chemistry data in the PLF at week 13 of the experimental period. The biomarkers μ-TP and μ-ALB were increased significantly in the male MWNT-7 group exposed to 3.2 mg/kg, while only μ-ALB was increased significantly in the 0.64 mg/kg male VGCF™-H group, and this was assumed to be the result of errors as the changes were not dose-dependent.

**Table 4:** Clinical chemistry in the PLF at week 13 of the experimental period

Test article	Total dose (mg/kg)	Male				Female			
			LDH	μ-TP	μ-ALB		LDH	μ-TP	μ-ALB
			(U/L)	(mg/dL)	(µg/mL)		(U/L)	(mg/dL)	(µg/mL)
Nontreatment	-	(5)	25 ± 39	29.8 ± 6.0	68.2 ± 9.9	(5)	2 ± 1	19.2 ± 5.5	50.2 ± 7.8
Control	0	(5)	7 ± 2	26.8 ± 2.5	63.7 ± 4.4	(5)	3 ± 3	17.1 ± 3.1	45.0 ± 5.0
MWNT-7	0.128	(5)	11 ± 7	31.4 ± 3.7	72.5 ± 6.5	(5)	4 ± 2	22.7 ± 1.3^#^	59.4 ± 2.3^#^
MWNT-7	0.64	(5)	11 ± 5	35.3 ± 8.0	76.3 ± 9.4	(5)	4 ± 1	23.7 ± 1.5^#^	57.5 ± 4.3^#^
MWNT-7	3.2	(1)	15 ± 6	39.6 ± 5.1^##^	83.9 ± 10.5^##^	(5)	8 ± 11	37.3 ± 23.8^#^	79.8 ± 38.7^#^
VGCF™-H	0.128	(5)	9 ± 3	30.5 ± 1.9	72.0 ± 5.0	(5)	4 ± 2	20.0 ± 3.4	53.1 ± 7.3
VGCF™-H	0.64	(5)	14 ± 12	31.5 ± 4.8	74.3 ± 9.5^#^	(5)	4 ± 2	19.3 ± 2.3	50.9 ± 7.5
VGCF™-H	3.2	(5)	7 ± 4	29.9 ± 2.9	69.2 ± 3.9	(5)	3 ± 1	19.6 ± 4.6	49.7 ± 8.7

Number in parentheses indicates the number of animals examined

The values indicate as mean ± S.D

Significantly different from the Control group; ^#^*p*< 0.05, ^##^*p*< 0.01

Table 5 shows the clinical chemistry data in the PLF at week 104 of the experimental period. No significant increase was observed for LDH, μ-TP, or μ-ALB in any of the male and female MWNT-7 and VGCF™-H groups. Many of the male animals in the group that received 3.2 mg/kg of MWNT-7 died by 104 weeks of the experiment, and the levels of these markers were therefore measured in only one rat. The results indicate that the pulmonary damage associated with VGCF™-H was lower than that for MWNT-7.

**Table 5:** Clinical chemistry in the PLF at week 104 of the experimental period

Test article	Total dose	Male				Female			
	(mg/kg)		LDH	μ-TP	μ-ALB		LDH	μ-TP	μ-ALB
			(U/L)	(mg/dL)	(µg/mL)		(U/L)	(mg/dL)	(µg/mL)
Nontreatment	–	(5)	9 ± 2	38.8 ± 8.9	81.3 ± 20.3	(5)	6 ± 4	48.5 ± 16.7	115.4 ± 35.7
Control	0	(5)	19 ± 20	66.5 ± 41.4	131.2 ± 62.4	(5)	5 ± 2	43.3 ± 7.2	103.7 ± 16.8
MWNT-7	0.128	(5)	11 ± 2	62.0 ± 29.0	117.4 ± 42.0	(5)	5 ± 1	46.8 ± 16.7	112.9 ± 38.8
MWNT-7	0.64	(5)	23 ± 17	59.6 ± 21.7	103.0 ± 18.1	(5)	8 ± 4	49.2 ± 29.6	106.2 ± 62.5
MWNT-7	3.2	(1)	46	257.1	307.3	(5)	32 ± 40	136.4 ± 129.8	192.0 ± 114.9
VGCF™-H	0.128	(5)	8 ± 2	40.4 ± 14.3	92.9 ± 27.6	(5)	6 ± 2	44.4 ± 21.7	105.0 ± 47.3
VGCF™-H	0.64	(5)	13 ± 9	58.1 ± 19.4	116.8 ± 26.4	(5)	11 ± 15	51.5 ± 20.7	118.8 ± 33.1
VGCF™-H	3.2	(5)	13 ± 6	81.8 ± 64.0	145.9 ± 63.3	(5)	10 ± 6	61.7 ± 25.5	142.3 ± 54.1

There was no significant difference between groups

Number in parentheses indicates the number of animals examined

The values indicate as mean ± S.D

### Distribution of carbon fibers

#### Amount of VGCF™-H and MWNT-7 fibers in the lung

To investigate lung toxicity relationships between the instillation of carbon fibers and lung toxicity such as inflammation and carcinogenicity, the lung burden of the carbon fibers was examined. Tables 6 and 7 show the amount of carbon fibers in the lungs of both male and female rats at weeks 13 and 104 of the experimental period. Table 6 shows the amount of carbon fibers in the lungs of each animal were observed to have increased in a dose-dependent manner by week 13 in the male MWNT-7 and VGCF™-H. The lung weight of males in both the MWNT-7 and VGCF™-H groups changed slightly with dose; however, the amount of carbon fibers per lung tended to be similar to the amount of carbon fibers administered. Interestingly, although the amount of carbon fibers in the lungs increased in a dose-dependent manner in both the male MWNT-7 and VGCF™-H groups, the amount of VGCF™-H observed was less than half that seen in the MWNT-7 group at week 104 of the experimental period in males that were subjected to 0.64 mg/kg. The amount of fibers in the lung could not be accurately measured in the male 3.2 mg/kg MWNT-7 group because many animals had died before week 104.

**Table 6:** Amount of carbon fibers in the lungs of male rats

Test article	Total dose		Week 13 of the experimental period		Week 104 of the experimental period		
	(mg/kg)		(μg/animal)	(μg/g lung)		(μg/animal)	(μg/g lung)
Control	0	(5)	–	–		–	–
MWNT-7	0.128	(5)	19.7 ± 2.50	15.5 ± 2.6	(5)	6.36 ± 1.60	4.0 ± 0.9
MWNT-7	0.64	(5)	132 ± 140	96.3 ± 8.0	(5)	73.9 ± 9.4	44.1 ± 8.6
MWNT-7	3.2	(5)	754 ± 55.0	458.5 ± 40.6	(0)	–	–
VGCF™-H	0.128	(5)	21.0 ± 2.60	16.2 ± 2.1	(5)	9.95 ± 1.8	6.3 ± 1.8
VGCF™-H	0.64	(5)	129 ± 17.0	101.4 ± 12.3	(5)	27.1 ± 6.2	16.6 ± 4.0
VGCF™-H	3.2	(5)	581 ± 61.0	422.1 ± 30.0	(5)	168.0 ± 154.0	106.4 ± 110.8

Number in parentheses indicates the number of animals examined

The values indicate as mean ± S.D

**Table 7:** Amount of carbon fibers in the lungs of female rats

Test article	Total dose	Week 13 of the experimental period				Week 104 of the experimental period		
	(mg/kg)		(μg/animal)	(μg/g lung)			(μg/animal)	(μg/g lung)
Control	0		–	–			–	–
MWNT-7	0.128	(5)	8.20 ± 1.38	9.0 ± 1.3	(5)		4.10 ± 1.17	3.5 ± 0.6
MWNT-7	0.64	(5)	55.6 ± 3.30	58.6 ± 3.4	(5)		40.7 ± 4.4	36.5 ± 4.8
MWNT-7	3.2	(5)	361 ± 48.0	308.6 ± 42.8	(5)		315.0 ± 49.0	251.4 ± 34.3
VGCF™-H	0.128	(5)	12.3 ± 1.60	13.3 ± 2.1	(4)		7.14 ± 0.99	6.5 ± 1.2
VGCF™-H	0.64	(5)	58.2 ± 13.5	64.8 ± 14.7	(5)		26.5 ± 3.1	24.8 ± 3.2
VGCF™-H	3.2	(5)	301 ± 34.0	323.3 ± 26.4	(5)		39.3 ± 20.3	33.7 ± 18.2

Number in parentheses indicates the number of animals examined

The values indicate as mean ± S.D

Table 7 shows the amount of carbon fibers and the lung weight of females at weeks 13 and 104 of the experimental period. The amount of carbon fibers in the lung was observed to increase in a dose-dependent manner by week 13, with equivalent amounts observed in the lungs of rats receiving different doses of MWNT-7 and VGCF™-H. The amount of carbon fibers in the lungs of females in the 3.2 mg/kg VGCF™-H group was less than 1/7 that of the MWNT-7 group at week 104.

These results suggested that the lungs were cleared off the VGCF™-H fibers more rapidly than the MWNT-7 fibers in both males and females.

Additional file 1: Tables S2 and S3 shows the results of converting the amount of carbon fiber in the lung to the surface area of the carbon fiber. The total surface area of carbon fibers in the lung was greater for MWNT-7 than for VGCF™-H in both sexes up to 13 weeks after instillation. At 104 weeks of instillation, the difference was even greater, VGCF™-H at 0.64 mg/kg in males was less than 1/4 that of MWNT-7, and VGCF™-H at 0.64 mg/kg and 3.2 mg/kg in females were less than 1/2 and 1/12 that of MWNT-7, respectively. These results are similar to Tables 6 and 7, suggesting that VGCF™-H has a lower biopersistence than MWNT-7.

#### Number of VGCF™-H and MWNT-7 fibers in the PLF

Table 8 shows the number of carbon fibers in the PLF of males and females at weeks 13 and 104 of the experimental period, which can be utilized to investigate the relationship between carcinogenicity and the number of carbon fibers in the pleural cavity. The numbers of MWNT-7 and VGCF™-H fibers in the pleural cavity were observed to increase in a dose-dependent manner, with less than 1/10 and 1/30 VGCF™-H fibers observe as compared to MWNT-7 groups at all doses for both male and female rats at week 13. Most importantly, the increase in the number of MWNT-7 and VGCF™-H fibers in the pleural cavity observed at week 104 of the experimental period was dose-dependent increases and the number of VGCF™-H fibers was less than 1/8 that seen in the MWNT-7 groups that received 0.128 and 0.64 mg/kg. The number of fibers in the PLF could only be measured in one animal in the 3.2 mg/kg male MWNT-7 group because most of the animals were dead by week 104 of the experimental period.

**Table 8:** Number of carbon fibers in the PLF

Test article	Total dose	Male				Female			
	(mg/kg)	(fibers/animal)				(fibers/animal)			
		Week 13		Week 104		Week 13		Week 104	
Control	0	–		–		–		–	
MWNT-7	0.128	1421 ± 772	(5)	4070 ± 2226	(5)	1445 ± 493	(5)	1338 ± 924	(5)
MWNT-7	0.64	4994 ± 3107	(5)	9134 ± 9059	(5)	4011 ± 2416	(5)	16,938 ± 14,546	(5)
MWNT-7	3.2	51,134 ± 17,273	(5)	6520	(1)	8835 ± 4166	(5)	8174 ± 3419	(4)
VGCF™-H	0.128	77 ± 70	(5)	144 ± 128	(5)	48 ± 66	(5)	99 ± 106	(5)
VGCF™-H	0.64	339 ± 239	(5)	1090 ± 549	(5)	93 ± 97	(5)	312 ± 253	(5)
VGCF™-H	3.2	1332 ± 224	(5)	5270 ± 2325	(5)	78 ± 72	(5)	2285 ± 1843	(5)

Number in parentheses indicates the number of animals examined

The values indicate as mean ± S.D

These results indicated that the carbon fibers were retained in the PLF for 104 weeks, and that the VGCF™-H fibers were less easily transferred from the lungs to the pleural cavity as compared to the MWNT-7 fibers.

### Changes in lung weights

No significant change was observed in the lung weight of the male MWNT-7 and VGCF™-H groups; however, the weight of the lungs from the 3.2 mg/kg female MWNT-7 group were found to have increased significantly at week 104 of the experimental period (Table 9). The lung weights of males in the 3.2 mg/kg MWNT-7 groups could not be measured because most of the animals in this group were dead by week 104 of the experimental period.

**Table 9:** Lung weights at week 104 of the experimental period

Test article	Total dose (mg/kg)	Male		Female	
		Lung weights (g)		Lung weights (g)	
Nontreatment	–	1.391 ± 0.381	(24)	0.940 ± 0.125	(16)
Control	0	1.328 ± 0.113	(21)	0.961 ± 0.083	(23)
MWNT-7	0.128	1.358 ± 0.121	(17)	0.986 ± 0.183	(18)
MWNT-7	0.64	1.403 ± 0.137	(4)	1.075 ± 0.288	(16)
MWNT-7	3.2			1.110 ± 0.046#	(4)
VGCF™-H	0.128	1.319 ± 0.063	(15)	1.094 ± 0.644	(18)
VGCF™-H	0.64	1.374 ± 0.206	(24)	0.951 ± 0.045	(21)
VGCF™-H	3.2	1.381 ± 0.084	(20)	0.955 ± 0.077	(21)

Number in parentheses indicates the number of animals examined

The values indicate as mean ± S.D

Significantly different from the Control group; ^#^*p* < 0.05

### Gross pathological examination

**Fig. 4:**
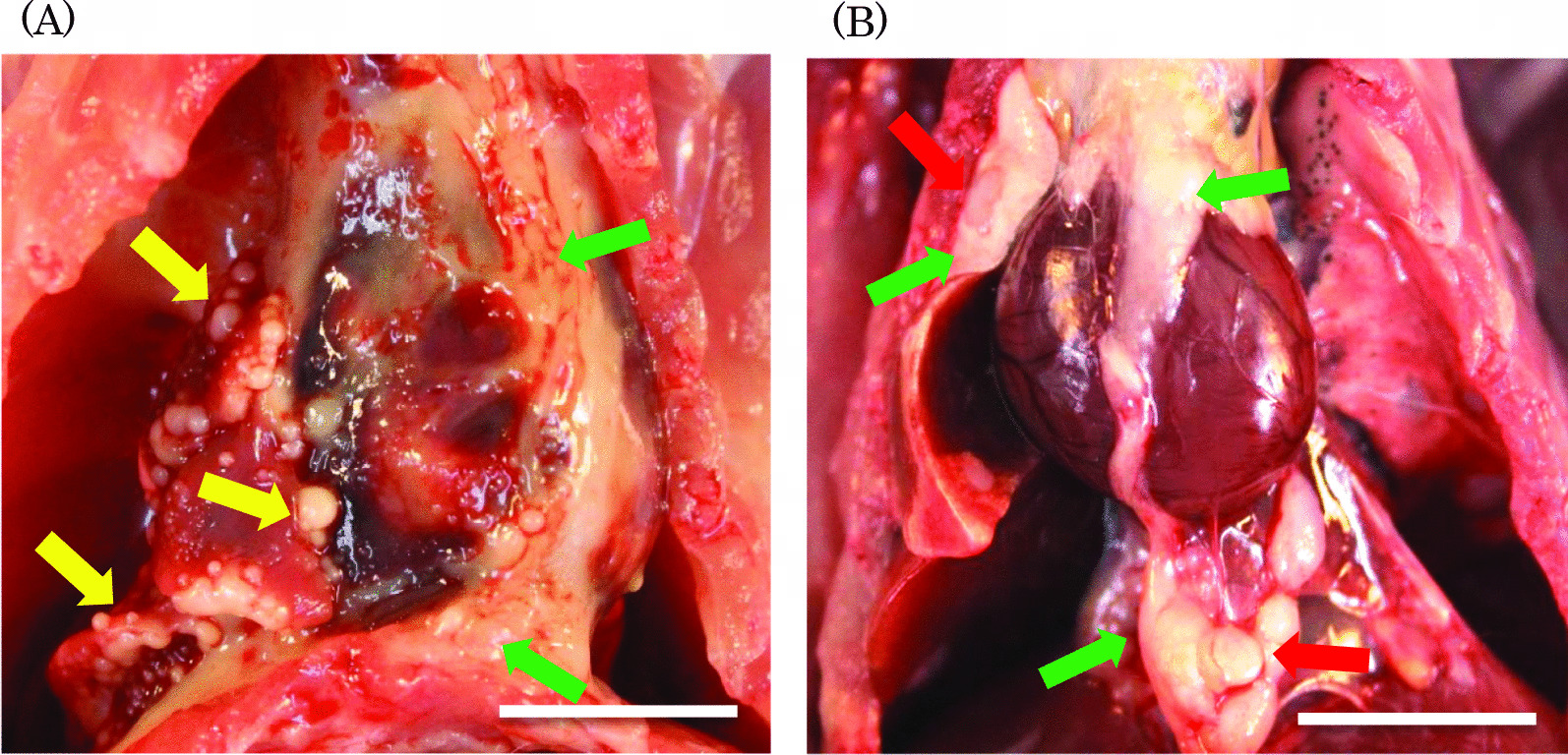
Representative gross pathological image of thoracic area in the male MWNT-7 group that died at week 45 of the experimental period (**A**) and an image of the male VGCF™-H group that died at week 60 of the experimental period (**B**). The small white granules (yellow arrows) and white nodule (green arrows) had spread around the lungs, and reached the heart epicardium, diaphragm, and thoracic wall (**A**). The white nodule (green arrows) and the swelling (red arrows) present around the lungs and heart (**B**). The scale bar indicates 10 mm

Gross pathological examination was performed on dead, euthanized, and animals surviving at week 104 of the experimental period. Most of the dead or euthanized animals in the MWNT-7 groups had nodules or small granules adhered to the thoracic wall, heart, or diaphragm, with clear/red fluid in their pleural or pericardial cavities. One image of a male rat in the MWNT-7 group that died in week 45 of the experimental period shows small white granules (Fig. 4A yellow arrows), white nodules (Fig. 4A green arrows), and black deposits in the thoracic area and the interior of the lung. The granules and nodules had spread around the lungs and reached the heart epicardium, diaphragm, and thoracic wall. One image of a male rat from the VGCF™-H group that died at week 60 shows white nodules (Fig. 4B green arrows) and swelling (Fig. 4B red arrows) around the lungs and the heart. These profiles were confirmed in animals that survived until week 104, male animals that died since week 46 of the experimental period, and in female MWNT-7 groups that died since week 66. Most importantly, these profiles were confirmed only in two animals, one of which was euthanized at week 60 and one in the male 3.2 mg/kg VGCF™-H group that died 102 weeks into the experiment.

### Histopathological examination

#### Neoplastic lesions

**Fig. 5:**
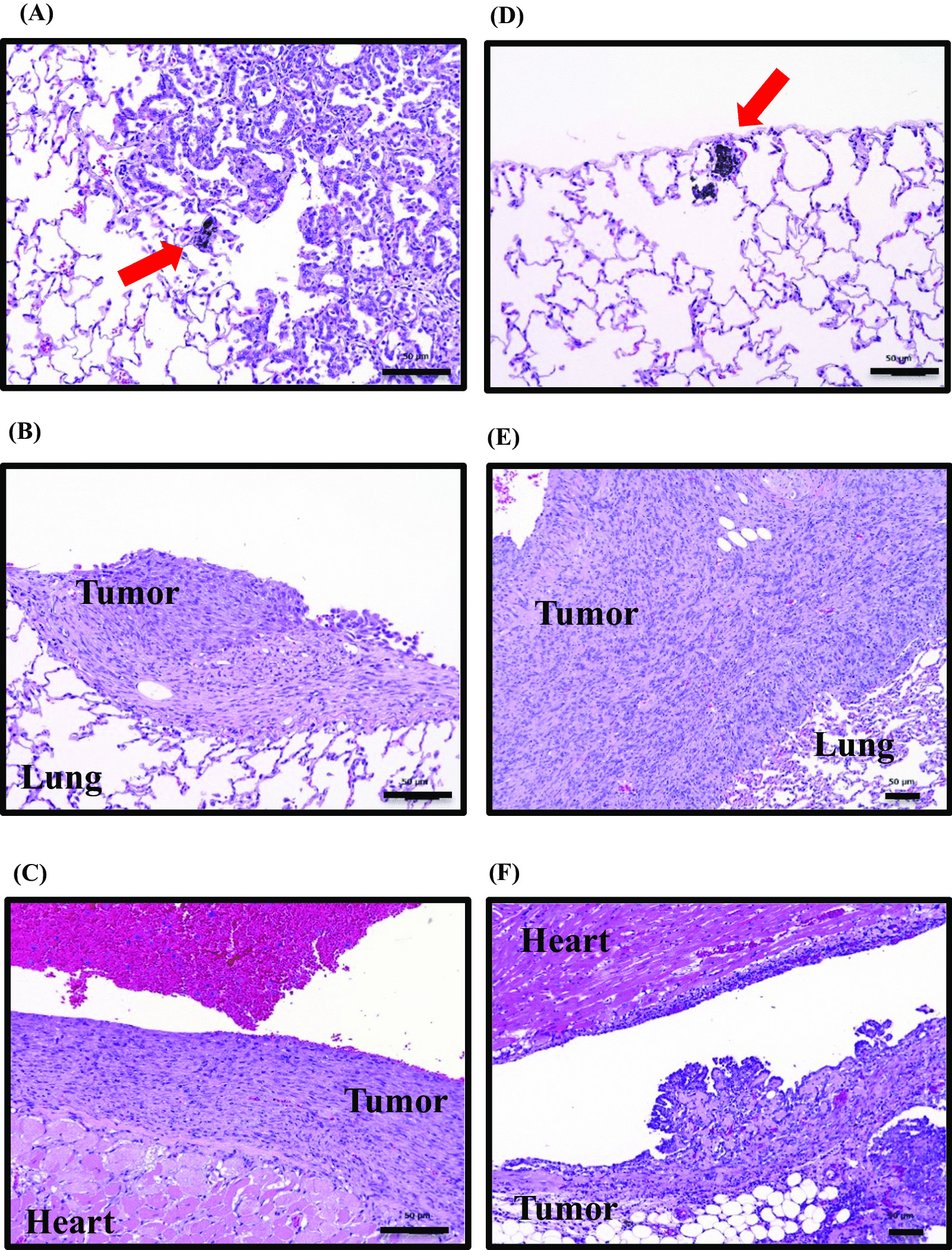
Representative histological images of the lung and heart in the MWNT-7 and VGCF™-H group. Deposition of MWNT-7 fibers (arrow in **A**) and the alveolar hyperplasia (**A**). **B**, **C** indicates the malignant mesothelioma in the MWNT-7 group. It indicates the deposition of VGCF™-H fibers (arrow in **D**) in the lung (**D**). **E**, **F** indicates the malignant mesothelioma in the VGCF™-H group. The scale bar indicates 50 μm

Table 10 shows details of the neoplastic lesions that were observed in the histopathology of dead, euthanized, and surviving rats 104 weeks after intratracheal instillation. No significant increase was observed in the incidence of adenoma, adenocarcinoma, and combined them in the lungs and bronchia of males or females in the MWNT-7 and VGCF™-H groups as a result of any dose. Adenosquamous carcinoma was observed in the lung/bronchia of one male and one female from the 3.2 mg/kg MWNT-7 groups, but none was observed at any dose in the VGCF™-H group. The incidences of malignant mesothelioma in the thoracic cavity 1 in 30 (3.3%), 18 in 30 (60.0%), and 37 in 39 (94.9%) in males that received 0.128, 0.64, and 3.2 mg/kg MWNT-7, and there was a significant increase in them compared to control group. However, the incidences in the thoracic cavity were 0 in 30 (0%), 0 in 30 (0%), and 2 in 30 (6.7%) in males that received 0.128, 0.64, and 3.2 mg/kg VGCF™-H, and there were not statistically differences from that of control group. Interestingly, the incidences of mesothelioma of the dead and euthanized rats were 1 in 12 (8.3%), 15 in 24 (62.5%), and 37 in 39 (94.9%) in the groups that received 0.128, 0.64, and 3.2 mg/kg of MWNT-7, respectively, while only 2 in 10 (20%) of the male rats that received 3.2 mg/kg in VGCF™-H group. This result suggested that malignant mesothelioma was developed early after carbon fiber instillation. The incidence of malignant mesothelioma in the thoracic cavity was 2 in 30 (6.7%), 1 in 30 (3.3%), and 22 in 30 (73.3%) in female rats that received 0.128, 0.64, and 3.2 mg/kg MWNT-7, and there was a significant increase in them compared to control group. No malignant mesothelioma was observed in any of the female VGCF™-H groups at any dose. The development of malignant mesothelioma in the thoracic cavity was dose-dependent increase in both male and female MWNT-7 groups. Most malignant mesothelioma had spread to the surface of the lungs (Fig. 5B for the MWNT-7 group, Fig. 5E for the VGCF™-H group), heart epicardium (Fig. 5C in MWNT-7, Fig. 5F in VGCF™-H group), thoracic wall, and diaphragm and had invaded each organ. Mesothelium hyperplasia of the heart increased significantly compared to control group in the male groups that received 0.64 mg/kg and 3.2 mg/kg and female groups that received 3.2 mg/kg MWNT-7 but was not observed in the VGCF™-H groups. Interestingly, the alveolar hyperplasia, bronchiolo alveolar hyperplasia, and mesothelium hyperplasia on the lung/bronchia increased significantly compared to control group in the MWNT-7 groups, but not in the VGCF™-H groups. The mesothelial hyperplasia of the diaphragm increased significantly compared to control group in both the 0.64 mg/kg and 3.2 mg/kg male and female MWNT-7 groups and the male 3.2 mg/kg VGCF™-H group but was not observed in any of the female VGCF™-H groups at any dose.

**Table 10:** Summary of histopathological findings for neoplastic lesions at 104-weeks of the experimental period

Test article	Male								Female							
	Nontreatment	Control	MWNT-7			VGCF™-H			Nontreatment	Control	MWNT-7			VGCF™-H		
Total dose (mg/kg)	–	0	0.128	0.64	3.2	0.128	0.64	3.2	–	0	0.128	0.64	3.2	0.128	0.64	3.2
No. of animals/group	30	30	30	30	39	30	30	30	30	30	30	30	30	30	30	30
*Lung/bronchial*																
Adenoma, bronchiolo-alveolar	0	4	1	0	1	2	0	0	1	0	3	0	0	1	0	2
Adenocarcinoma, bronchiolo-alveolar	0	0	0	0	0	0	0	1	0	0	0	1	0	0	0	0
Carcinoma, adenosquamous	0	0	0	0	1	0	0	0	0	0	0	0	1	0	0	0
Total adenoma and/or carcinoma	0	4	0	0	2	2	0	1	1	0	3	1	1	1	0	2
*Thoracic cavity*																
Mesothelioma, malignant	0	0	1	18##	37##	0	0	2	0	0	2	1	22##	0	0	0

There were no significant differences from the incidences of the other neoplastic lesions compared to control group in both the male and female MWNT-7 and VGCF™-H groups (Tables 11 and 12).

**Table 11:** Histopathological findings: summary of neoplastic (benign) or neoplastic (malignant) in male

Test article	Nontreatment	Control	MWNT-7			VGCF™-H		
Total dose (mg/kg)			0.13	0.64	3.2	0.13	0.64	3.2
Number of animals	30	30	30	30	39	30	30	30
*Heart*								
Schwannoma, intramural	0	1	0	0	0	0	0	0
Schwannoma, subendocardial	0	0	0	0	0	1	0	0
*Other lymph node*	[2]b	[3]b	[3]b	[4]b	[3]b	[3]b	[5]b	[4]b
Hemangioma	0	0	0	1	0	0	0	0
*Spleen*								
Fibroma	0	0	0	0	0	0	0	0
*Pituitary*	[12]b	[11]b	[10]b	[9]b	[3]b	[11]b	[12]b	[12]b
Adenoma, pars distalis	9	9	6	5	3	8	11	10
Adenoma, pars intermedia	1	0	0	0	0	0	0	0
Carcinoma, pars distalis	0	0	2	1	0	0	1	0
Craniopharyngioma, malignant	0	0	0	0	0	0	0	0
*Thyroid*	[10]b	[9]b	[9]b	[8]b	[1]b	[6]b	[7]b	[7]b
Adenoma, C-cell	6	7	8	5	1	6	4	6
Adenoma, follicular cell	0	1	0	0	0	1	0	0
Carcinoma, C-cell	2	1	1	3	0	1	1	2
Carcinoma, follicular cell	0	0	1	0	0	0	0	0
*Adrenal*	[3]b	[2]b	[1]b	[1]b	[1]b	[4]b	[3]b	[1]b
Ganglioneuroma, benign	0	0	0	0	0	0	0	0
Pheochromocytoma, benign	1	1	0	1	0	1	0	1
Pheochromocytoma, complex, malignant	0	0	0	0	0	1	1	0
Pheochromocytoma, malignant	2	0	1	0	0	0	0	0
*Nasal cavity*		[1]b				[1]b		
Adenocarcinoma		0				1		
*Tongue*					[1]b			
Carcinoma, squamous cell					1			
*Stomach*	[3]b	[3]b	[1]b	[2]b	[6]b	[1]b	[1]b	
Adenocarcinoma	1	0	0	0	0	0	0	
Leiomyosarcoma	0	0	0	0	1	1	0	
*Jejunum*		[1]b	[3]b	[1]b	[1]b		[1]b	
Gastrointestinal stromal tumor		0	0	0	0		0	
Leiomyosarcoma		0	1	0	0		0	
*Cecum*		[3]b	[3]b			[2]b		
Leiomyoma		0	1			0		
*Pancreas*	[1]b	[1]b		[2]b	[3]b		[4]b	[2]b
Adenoma, islet cell	0	0		0	1		0	0
Carcinoma, islet cell	0	1		0	0		2	1
*Liver*								
Adenoma, hepatocellular	3	0	1	0	0	2	0	0
Carcinoma, hepatocellular	0	0	1	0	0	0	1	0
*Kidney*								
Adenoma	0	1	0	0	0	0	0	0
Hemangioma	0	0	0	0	0	1	0	0
Carcinoma, transitional cell	0	0	0	0	0	1	0	0
*Urinary bladder*	[2]b	[1]b			[1]b	[2]b		
Papilloma, transitional cell	0	0			0	1		
*Testis*	[26]b	[27]b	[26]b	[23]b	[24]b	[27]b		[26]b
Adenoma, leydig cell	26	25	24	21	20	25	27	26
*Prostate*		[1]b	[1]b	[1]b	[1]b			
Adenoma		0	1	0	0			
Adenocarcinoma		0	0	0	0			
*Prep./ Clit. gland*				[3]b		[2]b	[3]b	[2]b
Adenoma				0		0	0	2
Adenocarcinoma				3		2	2	0
*Mammary gland*	[2]b	[1]b	[1]b	[3]b	[1]b	[1]b		[2]b
Fibroadenoma	2	0	1	0	0	1		1
*Thoracic wall*		[1]b	[3]b	[12]b	[36]b			[1]b
Osteosarcoma		0	1	0	0			0
*Skin/subcutis*	[7]b	[9]b	[6]b	[9]b	[2]b	[6]b	[11]b	[5]b
Fibroma	6	6	3	4	0	4	6	1
Keratoacanthoma	0	2	1	0	0	1	2	1
Lipoma	1	0	0	1	0	0	0	0
Papilloma, squamous cell	0	0	0	1	0	0	3	0
Trichoepithelioma	0	0	1	0	0	0	0	0
Carcinoma, squamous cell	0	2	0	0	0	0	0	0
*Skin/subcutis*								
Fibrosarcoma	0	0	0	1	1	0	2	1
Hemangiosarcoma	0	0	0	0	0	0	1	1
Malignant fibrous histiocytoma	0	0	0	1	0	0	0	0
Malignant schwannoma	0	0	0	1	0	0	0	0
Rhabdomyosarcoma	0	1	0	0	0	0	0	0
Sarcoma, NOS	0	0	0	0	0	0	0	1
*Zymbal's gland*		[1]b		[1]b	[2]b	[1]b		
Carcinoma		1		1	2	1		
*Brain*	[3]b	[5]b	[5]b	[2]b	[1]b	[3]b	[3]b	[3]b
Astrocytoma, malignant, low grade	0	0	0	0	0	1	0	0
Tumor, granular cell, malignant	0	0	0	0	0	0	0	1
Invasion (pituitary: carcinoma pars distalis)	0	0	2	1	0	0	0	0
*Abdominal cavity*	[1]b	[2]b	[2]b	[2]b	[4]b	[1]b	[3]b	
Mesothelioma, malignant	1	2	2	2	4	1	3	
*Pinna*					[1]b			
Neural crest neoplasm					1			
*All sites*	[4]b	[6]b	[4]b	[6]b	[2]b	[7]b	[5]b	[6]b
Histiocytic sarcoma	0	0	0	0	2	0	0	0
Large granular lymphocyte leukemia	4	4	4	6	0	7	4	4
Lymphocytic lymphoma	0	1	0	0	0	0	1	2
Myeloid leukemia	0	1	0	0	0	0	0	0
*Cranial bone*			[1]b					
Chondrosarcoma			1					
*Lumber vertebra*		[1]b						
Osteosarcoma		1						
*Sternum*								
Osteosarcoma								

Significantly different from the Control group; ^##^*p* < 0.01

^b^Numbers in square bracket are for animals examined microscopically

**Table 12:** Histopathological findings: summary of neoplastic (benign) or neoplastic (malignant) in female

Test article	Nontreatment	Control	MWNT-7			VGCF™-H		
Total dose (mg/kg)			0.13	0.64	3.2	0.13	0.64	3.2
Number of animals	30	30	30	30	30	30	30	30
*Heart*								
Schwannoma, intramural	0	0	0	0	2	0	1	0
*Pituitary*	[17]b	[21]b	[19]b	[16]b	[13]b	[21]b	[25]b	[21]b
Adenoma, pars distalis	8	12	15	10	7	13	16	10
Adenoma, pars intermedia	0	0	0	0	0	0	0	1
Carcinoma, pars distalis	2	0	1	1	2	1	4	2
*Thyroid*	[6]b	[3]b	[3]b	[2]b	[3]b	[5]b	[7]b	[5]b
Adenoma, C-cell	4	2	1	2	3	4	5	3
Carcinoma, C-cell	2	1	1	0	0	1	3	1
*Adrenal*			[3]b	[2]b	[1]b	[3]b	[1]b	
Adenoma, cortical			0	0	0	0	1	
Ganglioneuroma, benign			0	1	0	0	0	
Pheochromocytoma, complex, malignant			0	0	1	0	0	
Pheochromocytoma, malignant			1	0	0	0	0	
*Lung/bronchial*								
Epithelioma, non-keratinizing	0	0	0	0	1	0	0	0
*Stomach*	[1]b	[1]b	[4]b		[2]b	[3]b		[2]b
Papilloma, squamous cell	0	0	0		0	1		0
*Jejunum*	[1]b		[1]b	[1]b				[1]b
Leiomyosarcoma	0		0	1				0
*Cecum*	[1]b		[2]b					[1]b
Leiomyoma	1		0					0
Lipoma	0		1					0
*Colon*			[1]b			[1]b		
Leiomyosarcoma			0			1		
*Liver*								
Adenoma, hepatocellular	0	1	0	2	2	0	0	3
Carcinoma, hepatocellular	1	0	0	0	0	0	0	0
Cholangiocarcinoma	0	0	0	1	0	1	0	0
*Kidney*								
Hemangioma	0	0	0	0	1	0	0	0
Carcinoma	0	0	0	0	0	1	0	0
*Urinary bladder*	[1]b		[2]b		[2]b			
Carcinoma, transitional cell	0		0		0			
*Prep./ Clit. gland*	[4]b		[2]b	[5]b		[1]b	[3]b	
Adenoma	2		0	0		0	2	
Adenocarcinoma	1		2	5		1	1	
*Mammary gland*	[9]b	[10]b	[11]b	[8]b	[6]b	[14]b	[11]b	[8]b
Adenoma	0	0	1	1	0	1	2	2
Fibroadenoma	4	8	7	7	6	11	9	6
Fibroma	1	1	1	1	0	0	0	0
Adenocarcinoma	0	1	1	1	0	0	0	2
*Ovary*	[3]b	[1]b	[6]b	[2]b	[1]b	[5]b	[4]b	
Cystadenoma	0	0	0	0	0	1	0	
Luteoma	1	0	0	0	0	0	0	
Tumor, granulosa cell, benign	0	0	0	0	0	0	3	
*Uterus*	[8]b	[11]b	[9]b	[6]b	[3]b	[5]b	[8]b	[9]b
Leiomyoma	0	0	1	0	0	0	0	0
Polyp, endometrial stromal	3	5	3	1	1	4	3	6
Polyp, glandular	0	0	2	0	1	0	3	0
Adenocarcinoma	0	2	0	0	0	0	0	0
Leiomyosarcoma	0	0	0	1	0	0	0	0
Malignant schwannoma	0	0	1	0	0	0	0	0
Sarcoma, endometrial stromal	1	0	0	0	0	0	0	0
*Skin/subcutis*	[3]b	[1]b	[3]b	[1]b	[1]b	[3]b	[2]b	[3]b
Adenoma, sebaceous cell	0	0	0	0	0	0	1	1
Fibroma	1	1	0	0	0	0	0	1
Keratoacanthoma	0	0	0	0	0	0	1	0
Malignant fibrous histiocytoma	0	0	1	0	0	0	0	0
Malignant schwannoma	0	0	0	0	1	0	0	1
Osteosarcoma	0	0	0	1	0	0	0	0
*Brain*	[5]b	[2]b	[6]b	[2]b	[4]b	[10]b	[10]b	[8]b
Invasion (pituitary: carcinoma pars distalis)	2	0	0	0	2	1	1	2
*Spinal cord*	[1]b	[2]b	[1]b					
Astrocytoma, malignant, high grade	0	0	1					
Astrocytoma, malignant, low grade	0	1	0					
*Abdominal cavity*			[1]b		[1]b			
Mesothelioma, malignant			0		1			
Sarcoma, NOS			1		0			
*Oral cavity*	[1]b							
Carcinoma, squamous cell	1							
*Trigeminal nerve*						[1]b		
Neurofibrosarcoma						1		
*All sites*	[6]b	[4]b	[6]b	[8]b	[1]b	[7]b	[6]b	[5]b
Histiocytic sarcoma	0	0	1	0	0	0	0	0
Large granular lymphocyte leukemia	4	3	5	4	1	5	4	5
Lymphocytic lymphoma	2	1	0	4	0	2	2	0
*Site unknown*						[1]b		
Endocrine tumor						1		

Significantly different from the Control group; ^##^*p* < 0.01

^b^Numbers in square bracket are for animals examined microscopically

#### Non-neoplastic and pre-neoplastic lesions

Table 13 exhibits the non-neoplastic lesions and pre-neoplastic lesions obtained from the dead, euthanized, and surviving rats via histopathology at 104 weeks following intratracheal instillation. Deposits comprising fibers and inflammatory cells that infiltrated the lungs and bronchia increased significantly compared to control group in both the male and female MWNT-7 and VGCF™-H groups. Pleura fibrosis, chronic inflammation, granuloma formation on the lung/bronchia, and pleural fibrosis in the diaphragm increased significantly compared to control group in the MWNT-7 groups, but not in the VGCF™-H groups.

**Table 13:**
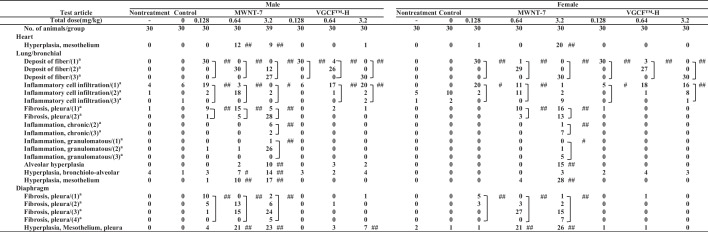
Summary of histopathological findings for non-neoplastic or pre-neoplastic lesions at 104-weeks of the experimental period

Significantly different from the Nontreatment group; **p < 0.01

Significantly different from the Control group; ^#^*p* < 0.05, ^##^*p* < 0.01

^a^Numbers in parenthesis indicate the grades of lesion: (1) minimum, (2) Slight, (3) Moderate, (4) Marked, (5) Severe

## Discussion

This study is a comparison of the carcinogenicities of the two different types of vapor grown carbon fibers VGCF™-H and MWNT-7, which were administered to rats via intratracheal instillation. Rats were instilled with the vehicle, MWNT-7, and VGCF™-H at doses of 0, 0.016, 0.08, and 0.4 mg/kg (total doses of 0, 0.128, 0.64, and 3.2 mg/kg) once a week for 8 weeks. Animals were then observed over a period of 2 years, following the first instillation and sacrificed after the observation period. Both MWNT-7 and VGCF™-H fibers led to an inflammatory response in the PLF, and carbon fibers were transferred to the pleural cavity; however, the magnitude of the inflammatory response and the number of carbon fibers observed were lower in the VGCF™-H groups than the MWNT-7 groups. Both MWNT-7 and VGCF™-H induced fibrosis and inflammatory cell infiltration in the lung; however, alveolar macrophage aggregation, pleural fibrosis, chronic inflammation, and granuloma formation on the lung and the bronchia were much milder in the VGCF™-H groups compared to the MWNT-7 groups. In particular, the incidence of malignant mesothelioma was much milder in the VGCF™-H groups compared to the MWNT-7 groups as the trend of these other toxicity in the lung and pleural, suggesting an association between the carbon fibers and the development of malignant mesothelioma.

The instillation of MWNT-7 clearly induced malignant mesothelioma in the pleural cavity at all doses. On the other hand, malignant mesothelioma was observed in only two cases for males that received the highest dose of VGCF™-H, and was not observed in any females that received VGCF™-H. Moreover, the MWNT-7 groups began to die earlier in the experiment than those in the VGCF™-H groups, and many of the animals that died or were euthanized in week 104 of the experimental period had developed malignant mesothelioma that had already spread into the thoracic cavity. This suggested that the malignant mesothelioma developed early after the initial instillation of the MWNT-7 fibers and was a direct cause of death. In addition, the incidence of malignant mesothelioma induced by carbon fibers was higher in males than in females. Carbon fiber dosage was calculated per animal weight. However, males and females weigh were differently, with males receiving a higher carbon fiber intake per serving than females. We believe that the difference in dosage between individuals affects the incidence of malignant mesothelioma.

**Fig. 6:**
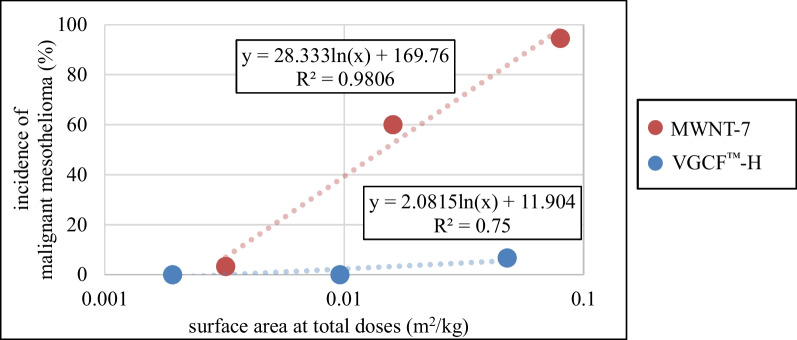
Correlation between surface area at total doses and the incidence of the malignant mesothelioma. The total dose of 0.128, 0.64, 3.2 mg/kg were a surface area of 0.0032, 0.016, and 0.08 m^2^/kg for MWNT-7, a surface area of 0.00192, 0.0096, and 0.048 m^2^/kg for VGCF™-H

In this study, we compared the mass of carbon fibers, but when comparing carbon fibers with different fiber lengths or fiber diameters, surface area may be used as a basis. The surface of carbon fiber had been known to relate to reactions and dispersion in organisms. Therefore, it has been reported that comparisons based on surface area rather than dose are more effective for demonstrating differences in toxicity of carbon fibers with the same material and similar shape. If the difference in incidence of malignant mesothelioma between MWNT-7 and VGCF™-H was due to their shape (size of fibers such as thickness), we expected that that depends on the surface area regardless of the type of carbon fiber. Therefore, we evaluated the incidence of malignant mesothelioma in MWNT-7 and VGCF™-H by surface area (Fig. 6). Total surface areas of 0.128, 0.64 and 3.2 mg/kg total doses were 0.0032, 0.016 and 0.08 m^2^/kg for MWNT-7 and 0.00192, 0.0096 and 0.048 m^2^/kg for VGCF™-H, respectively. We also showed that VGCF™-H had a lower incidence of malignant mesothelioma than MWNT-7, even when comparing total surface area corresponding to total dose. The incidence of malignant mesothelioma did not depend on the carbon fiber surface area, suggesting that factors other than carbon fiber surface area (stiffness, length, etc.), including the respective properties of VGCF™-H and MWNT-7 was considered to influence the difference in toxicity of carbon fibers. Interestingly, there was a linear correlation between the logarithmic distribution of the total surface area of MWNT-7 and the incidence of malignant mesothelioma, with a total surface area that did not develop malignant mesothelioma of 0.0025 m^2^/kg (as a total dose of 0.100 mg/kg). The total surface area of VGCF™-H that did not develop malignant mesothelioma was 0.0096 m^2^/kg, and VGCF™-H　also showed a lower incidence of malignant mesothelioma than MWNT-7 in this point.

Whole-body inhalation is useful for evaluating the lung toxicity of airborne materials that are likely to induce adverse effects in the lung. Recent studies that evaluated the lung toxicity of MWNT-7 by using whole-body inhalation performed at 0.2, 1, and 5 mg/m^3^ over 13 weeks showed persistent lung toxicity at 1 mg/m^3^ in rodents [11]. However, it has also been reported that rats exposed to 0.54, 2.5 and 25 mg/m^3^ of VGCF™-H via whole-body inhalation over 90 days showed a detectable accumulation of extrapulmonary fibers with minimal inflammation at 25 mg/m^3^ [10]. Furthermore, in a sub-chronic study, the administration of MWNT-7 or VGCF™-H by intratracheal instillation induced pulmonary toxicity with inflammation and fibrosis in a dose-dependent manner, with less lung toxicity associated with VGCF™-H than MWNT-7 [12]. The results of these studies and the present study indicate that the lung toxicities of MWNT-7 and VGCF™-H are dose-dependent increases, and that the lung toxicity of VGCF™-H is lower than that of MWNT-7 when administered by both whole-body inhalation and intratracheal instillation. Therefore, the intratracheal instillation method can be used for screening or toxicity ranking, and the effects of the lung toxicity, such as carcinogenicity or chronic toxicity of the extrapulmonary fibers, can be revealed using either method. Moreover, the use of reference material such as MWNT-7 may predict chronic toxicity and carcinogenicity for test material in the lung in sub-chronic studies.

The amount of carbon fiber in the lungs was equivalent in the VGCF™-H and MWNT-7 groups at 13 weeks into the experimental period, with lower amount of fibers observed in the VGCF™-H groups than the MWNT-7 groups at 104 weeks into the experimental period. A higher number of carbon fibers was observed in the PLF of the MWNT-7 group than that of the VGCF™-H group at both 13 and 104 weeks into the experimental period. These results indicate that VGCF™-H fibers are cleared more readily from the thoracic cavity than MWNT-7 fibers. Hojo et al. reported that a translocation of fibers through the visceral pleura could take place because of increases in pulmonary interstitial pressure as result inflammatory and edematous change. This report showed that increased pulmonary interstitial pressure caused carbon fibers to migrate from the lungs through the pulmonary pleura and into the thoracic cavity [15]. In addition, Hojo et al. suggested that pulmonary pleura edematous was more pronounced with repeated intratracheal instillation than with whole-body inhalation, and increased carbon fiber transition from the lungs into the thoracic cavity as a response specific to intratracheal instillation. However, 13 weeks after intratracheal administration of VGCF™-H, pulmonary pleura was hardly inflamed (3.2 mg/kg) in our previous study [12]. In addition, pulmonary pleural inflammation could not be observed because 2 years had passed since administration in this study, but fibrosis indicating long-term pulmonary pleural edema and inflammation was almost never observed in the VGCF™-H group.

Based on these results, we considered that one of the reasons for the lower number of fibers in the thoracic cavity in the VGCF™-H group compared to the MWNT-7 group is that the incidence of pleural translocation is less in the VGCF™-H group. Moreover, though in the whole-body inhalation, carbon fibers are mainly cleared from the lungs via lymphatic vessels, in addition to that route, intratracheal instillation was suggested that the transfer of carbon fibers from the lungs into the pleural cavity due to pulmonary pleural inflammation (edematous change) further clarified the mesothelioma-inducing potential of the administered carbon fibers. We also considered that there is an effect of carbon fiber length on the degree of pulmonary pleural inflammation. Anja Schinwald and Ken Donaldson reported that persistent pleural inflammation induced, because silver nanowires longer than 10 μm could not be completely phagocytosis by macrophages, and frustrated phagocytosis (a phenomenon in which long fibers destroy cells that phagocytize them) was induced, by visualizing method of the interaction between silver nanowires and pleural inflammatory cells [16]. VGCF™-H fibers used in our study show fewer fibers longer than 10 μm compared to MWNT-7 fibers (Additional file 1: Fig. S1). We suggested that VGCF™-H fibers are less likely to induce frustrated phagocytosis in macrophages than MWNT-7 fibers, and that it was one of factors which the degree of chronic inflammation, transition of VGCF™-H fibers into the thoracic cavity, and the incidence of mesothelioma. Furthermore, Donaldson et al. reported that biopersistent substances in the parietal cavity are transported through pores called stomata and excreted to the lymph nodes, and that fibrous substances with a large aspect ratio cannot pass easily through the stomata [17]. In this study, MWNT-7 induced hyperplasia of the diaphragm pleura, and it is possible that the structure of the parietal pleura was altered, thereby inhibiting the function of the stomata. In addition, VGCF™-H did not cause diaphragm pleural hyperplasia except for the highest dose in males, suggesting that VGCF™-H cleared the thoracic cavity faster than MWNT-7. These reports and results in this study suggested that the difference in the number of MWNT-7 and VGCF™-H fibers in the PLF in this study was related to both transition from the lung through the edematous pleura into the thoracic cavity and excretion from the thoracic cavity via the stomata. Moreover, Murphy et al. reported that the inflammatory response, fibrosis, and granuloma formation in several organs differed depending on the length, thickness, and shape (tangle or straight) of the carbon fiber, and that this difference in biological response caused that longer fibers clog the stomata and inhibit the clearance of carbon fibers from the thoracic cavity [18]. Interestingly, Murphy et al. reported that MWNT-7 clogged the stomata and caused a strong inflammatory response on the tissue surface. The smaller aspect ratio of the VGCF™-H fibers means that they can be cleared more rapidly from inside the thoracic cavity, and that MWNT-7 fibers are cleared from the pleural cavity with some difficulty. This may explain the early development of malignant mesothelioma and the higher rates of mortality observed in the MWNT-7 groups. On the other hand, it is unclear how the carbon fibers are transferred from the lung to the pleural cavity, and even two different straight type fibers for vapor grown carbon fibers produced different numbers of fibers in the lungs at week 104 of the experimental period. These results suggest that physical properties such as the aspect ratio affect the transition to the pleural cavity.

Previous studies have reported that some carbon fibers that are inhaled into the lung are transferred from the lung to the PLF, where they induce mesothelial hyperplasia [4] and malignant mesothelioma [5]. In this study, malignant mesothelioma occurred in the MWNT-7 and presumably the VGCF™-H groups, but the incidence was much higher in the MWNT-7 groups and more carbon fibers were observed in the pleural cavities of individuals from the former groups. It has been reported previously that the long-term inhalation of carbon fibers not only deposits carbon fibers in the lung, but also induces alveolar fibrosis and hyperplasia by transferring carbon fibers to the thoracic cavity [12], which is supported by the results of this study. The long-term inhalation of carbon fibers may therefore result in the development of malignant mesothelioma in the lung.

In contrast to the development of malignant mesothelioma, the intratrachial instillation of VGCF™-H and MWNT-7 almost certainly did not lead to lung adenoma or adenocarcinoma. In a previous report that showed lung carcinoma at exposures of 0.2 mg/m^3^ and 2 mg/m^3^ in male and female rats in a 2 years whole-body inhalation study of MWNT-7, the rats did not develop malignant mesothelioma [4]. Based on the correlation between lung deposition and the exposure dose shown by Kasai et al., the rat lung deposition of MWNT-7 fibers in our study was calculated as the exposure dose by whole-body inhalation. The lung deposits associated with the intratracheal instillation of 0.128 and 0.64 mg/kg MWNT-7 in male rats at week 104 of the experimental period were 0.007 and 0.082 mg/m^3^ when converted to whole-body inhalation doses, while female rats received with 0.128, 0.64, and 3.2 mg/kg MWNT-7 showed lung deposits of 0.007, 0.071, and 0.546 mg/m^3^ by whole-body inhalation at the same point in time. These results suggest that an insufficient amount of carbon fibers is deposited in the lungs to cause lung cancer. On the other hand, in our study, 4070 and 1338 MWNT-7 fibers were observed in the PLF of male and female rats exposed to 0.128 mg/kg MWNT-7, respectively, whereas Kasai et al. reported 1468 and 847 MWNT-7 fibers in the PLF of male and female rats, respectively, even at the maximum exposure of 2 mg/m^3^. This report suggests that the whole-body inhalation method might not have transferred sufficient fiber into the thoracic cavity to cause mesothelioma. This consideration is supported by a report by Fukushima et al., the results of which suggest that lung cancer was not observed in our study because intratracheal instillation administers a large amount of carbon fibers at the start of the experiment, and that large numbers of the fibers are quickly transferred into the thoracic cavity. In contrast, carbon fibers accumulate gradually in the lungs when administered by whole-body inhalation, meaning that a long time is required for an equivalent dose to that via intratracheal instillation to be reached [19]. In addition, it has been reported that rodents have a different nasal structure than humans, with a significantly higher nasal cavity surface area per body weight [20], meaning that the carbon fibers do not enter the lungs easily by whole-body inhalation. Carcinogenicity studies that include whole-body inhalation and intratracheal instillation may have different target organs, such as the lungs or thoracic cavity. On the other hand, in the recent whole-body inhalation study, the inhalation of MWNT-7 did not lead to malignant mesothelioma, although lung pleura hyperplasia was developed [4]. In addition, 5270 fibers were obtained from the PLF of the male 3.2 mg/kg VGCF™-H group with only 2 cases of mesothelioma observed, while 2285 fibers were obtained from the female 3.2 mg/kg group, with no mesothelioma observed. This result suggests that thousands of carbon fibers might be required to be present within the thoracic cavity as one of several conditions for the development of mesothelioma. If an animal is exposed to carbon fibers via whole-body inhalation for more than 2 years, the development of malignant mesothelioma may be observed because more fibers are transferred into the thoracic cavity. The results obtained for pulmonary toxicity, especially malignant mesothelioma, differ for the whole-body inhalation method and the intratracheal instillation method [4, 5, 12, 21]. Moreover, the current occupational exposure limits for workers are most often assessed using the whole-body inhalation method. However, the administration of MWNT-7 by whole-body inhalation does not lead to malignant mesothelioma. Therefore, it does not know if the mesothelioma, lung adenoma, or adenocarcinoma develop in 2 year carcinogenicity studies that uses whole-body inhalation to instill VGCF™-H fibers. It has been reported that the intratracheal instillation method can be used for the hazard identification and ranking of test substances. In order clearly to evaluate the carcinogenic potential of VGCF™-H, it is necessary to conduct a study using the whole-body inhalation method by VGCF™-H in addition to the results of this study. Hojo et al. reported that the lung burden peak of the carbon fiber in the lung differs between the whole-body inhalation method and the intratracheal instillation method, and that this affects the development of lung cancer and malignant mesothelioma [15]. It has been reported that the intratracheal administration method and the whole-body inhalation method are likely to develop malignant mesothelioma and lung adenocarcinoma, respectively. Hojo et al. adjusted the duration of administration to make the lung burden during intratracheal instillation similar to whole-body inhalation, resulting in the development of not only malignant mesothelioma but also lung adenocarcinoma with intratracheal instillation. These results suggest that temporal differences in carbon fiber exposure to the lung may cause differences in the incidence of lung adenocarcinoma and malignant mesothelioma. The results of this study support those reports and suggest that not only MWNT-7, which has been reported to be carcinogenic by intratracheal instillation, but also other carbon fibers have similar tendencies. Intratracheal instillation may be effective in assessing the potential development of malignant mesothelioma.

Sakamoto et al. conducted intraperitoneal administration studies of seven different multi-walled carbon nanotubes (MWCNTs) with iron contents ranging from ≤ 0.1 to 59 μg/mg CNTs in rats to evaluate the developmental potential of MWCNTs for malignant mesothelioma. In that report, there was little correlation between iron contents and incidence of malignant mesothelioma, suggesting that fiber shape (long and relatively thick fibers (≧50 nm)) was a critical factor in the development of that [22]. In the present study, the iron contents of MWNT-7 and VGCF™-H were 4200 ppm (4.2 μg/mg) and 9.7 ppm (0.0097 μg/mg), which are within the range of the study by Sakamoto et al. Therefore, it was considered unlikely that the iron content of each carbon fiber would affect the development of malignant mesothelioma. Our results also supported Sakamoto's report, as the incidence of malignant mesothelioma was higher in MWNT-7, which has more long fibers than VGCF™-H.

In our study, adenosquamous carcinoma, a rare malignant tumor, was found in 1 male and 1 female from the 3.2 mg/kg MWNT-7 groups. A previous report described the development of adenosquamous carcinoma in a 2 year carcinogenicity study that was performed using the whole-body inhalation of indium tin oxide (ITO) by rats, which is particulate matter with an average diameter of 3.5 μm [23]. This indicates that the development of adenosquamous carcinoma may result from exposure to MWNT-7. Although MWNT-7 did not cause lung adenoma and adenocarcinoma, the results of this study suggest that it may have carcinogenic potential in the lung because the administration of MWNT-7 led to alveolar hyperplasia and adenosquamous carcinoma.

## Conclusions

Under the conditions of this study, MWNT-7 showed clear carcinogenic properties in both male and female rats. There was also equivocal evidence of carcinogenic potential for VGCF™-H in male rats at the highest dose; however, this was no true for females.

Differences in the carcinogenicities of the two different carbon fibers were considered to be due to the number of carbon fibers in the pleural cavity. Thus, the carcinogenic activity of VGCF™-H is clearly lower than that of MWNT-7.

## Methods

### Preparation of the test materials

Both VGCF™-H (Showa Denko K.K., Japan) and MWNT-7 (Bussan Nanotech Laboratories, Inc., Japan) were dispersed in the same method as the sub-chronic tests in [12], as described below. Both materials were dispersed in saline solution containing 0.3% w/v Kolliphor P188 (KP188) (Sigma-Aldrich Japan Ltd., Japan) by a tabletop ultrasonic machine (M1800-J, Emerson Japan Inc., Tokyo, Japan); MWNT-7 was dispersed under deaeration (Vacuum Pump V-700, Japan Buchi Co., Ltd., Japan). The suspensions were sonicated with a probe-type ultrasonic generator (UD-201, TOMY SEIKO CO., LTD., Japan) and then dispersed in a wet dispersion system (HJP-25001, SUGINO MACHINE LIMITED., Japan) to prepare solutions at the correct dosage. The prepared solutions were stored in the refrigerator until instillation, and both vehicle and VGCF™-H solution were redispersed for 10 min before intratracheal instillation with a tabletop ultrasonic machine (M1800-J, Emerson Japan Inc., Japan) and then mixed in a vortex mixer for several seconds. The MWNT-7 solution was deaerated and redispersed for 1 min using a vacuum pump (Vacuum Pump V-700, Japan Buchi Co., Ltd., Japan) and a tabletop ultrasonic machine, redispersed for a further 9 min without deaeration, and then the vessel was shaken and gently stirred. All dosing solutions were used within 1 h of redispersion and the solutions were gently mixed just before instillation to produce a homogeneous solution.

### SEM

Test material solutions were diluted 100-fold with deionized water and filtered through a membrane filter (Whatman Nuclepore Track-Etch Membrane 111106 PC, Florham Park, USA) for observation with an electron microscope (JSM-7000F, JEOL Ltd., Japan). This membrane filter was subjected to plasma coating with osmium and observed with an electron microscope at an acceleration voltage of 5 keV.

### Characterization of the test materials

The average hydrodynamic diameter of fibers in 0.4 mg/mL solution of saline containing 0.3% w/v KP188 was measured using DLS (ELSZ-2000S, Otsuka Electronics Co., Ltd., Japan) at 25 °C. The hydrodynamic diameters obtained were the average of 8 measurements. The average hydrodynamic diameters did not change before and after passing through the microsprayer aerosolizer.

Iron contents of MWNT-7 and VGCF™-H fibers were determined by X-ray fluorescent analysis (RIGAKU RIX2100 and RIGAKU ZSX Primus II, Rigaku Corporation, Japan).

### Animals and husbandry

Eight-week-old male and nine-week-old female pathogen-free F344/DuCrlCrlj rats were obtained from Charles River Laboratories Japan, Inc. (Kanagawa, Japan). The animals were housed in a barriered-system animal room under controlled conditions (temperature, 22 ± 3 °C; humidity, 55 ± 15%; 12-h light–dark cycle) and were given the pellet diet CRF-1 sterilized with 30 kGy gamma irradiation (Oriental Yeast Co., Tokyo, Japan) and water ad libitum. After 14 days for male rats and 8 days for female rats, a quarantine and acclimation period were conducted after which the 10 week-old rats were randomized by body weight and assigned to groups (with 40 rats each in the untreated and the vehicle groups, and 50 in each of the VGCF™-H and MWNT-7 groups) on the day before the initial instillation. No significant differences in the average body weights were observed between the groups at the commencement of the study, as measured by the Bartlett and Tukey tests. In addition, no abnormalities were observed in the general condition of the animals during the quarantine period.

The study was approved by the Animal Experimental Committee at the DIMS Institute of Medical Science, Inc., and conducted in accordance with the “Law for the Humane Treatment and Management of Animals” (Law No. 46, May 2014), “Standards Relating to the Care and Management of Laboratory Animals and Relief of Pain” (Notice No. 84 of the Ministry of the Environment, September 2013), “Basic policies for the conduct of animal experiment in academic research institutions under the jurisdiction of the Ministry of Health, Labor, and Welfare” (Notice No. 0220-1 of the Ministry of Health, Labor and Welfare, February 2015), “Guidelines for Proper Conduct of Animal Experiments” (Science Council of Japan, June 2006), and “Standards for Care and Use of Laboratory Animals of DIMS Institute of Medical Science, Inc.” (June 1, 2016). This study was also conducted in accordance with GLP standards with reference to OECD TG451 [24].

### Experimental design and treatment of intratracheal instillation

Animal handling during and after the intratracheal instillation was performed as described previously [5, 25]. Briefly, rats were placed under isoflurane anesthesia using the NARCOBIT-E for small laboratory animals (Natsume Seisakusho Co., Ltd., Tokyo, Japan), and the instillation of the test material solution was performed intratracheally with a DIMS-type microsprayer aerosolizer (for rats) that was connected to a 1-mL disposable syringe (OSAKA CHEMICAL Co., Ltd., Osaka, Japan). This instillation method was the same as trans-tracheal intrapulmonary spraying (TIPS) [5, 13]. The instillation of MWNT-7 and VGCF™-H was performed once a week for 8 weeks (8 times in total). The single doses for this study were set at 0 (control), 0.016, 0.08, and 0.4 mg/kg body weight with total doses set at 0.128, 0.64, 3.2 mg/kg body weight with reference to the 13-week subchronic toxicity study by the intratracheal instillation of VGCF™-H and MWNT-7 previously reported [12]. The volume of solution used in instillation was 2 mg/kg and was calculated for each individual animal based on the body weight at the time of instillation. The animals in the control group were instilled with vehicle solution and the nontreatment group did not undergo either isoflurane anesthesia or insertion of the microsprayer aerosolizer. Animals were then observed without further treatment until each sacrifice timepoint.

### General observation, body weight, and examination of the animals

The general physical condition of all rats was checked three times on the day of intratrachial instillation; once immediately before and after instillation and once in the afternoon. All rats were observed twice per day until the end of the experimental period, except on instillation days.

All animals were individually weighted on the day of instillation and then weekly until the end of the experimental period. The body weight was also measured at the end of this study.

### Collection of pleural lavage fluid (PLF)

At 13-week and 104-week of the experimental period, 5 animals from each group were placed under deep isoflurane anesthesia and exsanguinated from the abdominal aorta. After blood collection, PLF was collected by a previously reported method [9].

### Analysis of inflammatory cells and clinical chemistry in PLF

The residual PLF cell pellets were resuspended in 1 mL of sterilized buffered physiological saline and processed for WBC and differential leukocyte counts using an automatic multi-item blood cell analyzer (XT-2000i, Sysmex Corporation, Hyogo, Japan). The supernatants were analyzed for alkaline phosphate (ALP), lactate dehydrogenase (LDH), protein concentration (total protein), and albumin (ALB) using an automatic analyzer (Hitachi 7070, Hitachi, Ltd., Tokyo, Japan). Interleukin 8 (IL-8), a marker of neutrophil migration factor [26, 27], was measured by an absorption reader (Model: Sunrise Rainbow RC, Tecan Japan Co., Ltd). The cell pellet was fixed by suspension in buffered 4% paraformaldehyde at 4 °C overnight, then centrifuged at 1000 rpm for 10 min at 4 °C. The pellet was washed with saline and centrifuged at 1000 rpm at 4 °C for 10 min. Sodium alginate (0.5 mL of 1%) and 20 μL of 1 M CaCl_2_ was added to the pellet, and the pellet was stored in 80% ethanol prior to embedding in paraffin and processing for histopathology.

### Measurement of number of MWNT-7 and VGCF™-H fibers in the lung and PLF

At weeks 13 and 104 of the experimental period, five animals that were not subjected to PLF in each of groups were exsanguinated from the abdominal aorta under deep isoflurane anesthesia. After blood collection, lung tissue, including trachea and bronchi, were weighed, and then preserved in a 10% buffered formalin solution for lung burden analysis. To measure the amount of VGCF™-H and MWNT-7 in the lung and PLF, fixed lung tissues and PLF sample were sent to the Japan Bioassay Research Center, Japan Organization of Occupational Health and Safety (Kanagawa, Japan).

For measurement of carbon fibers, 5 mL in PLF was agitated with a touch mixer and 1 mL was collected, then centrifuged at 12,000 rpm for 10 min. The supernatant was removed and the pellet was digested according to the method of Kohyama et al. [28]. A polycarbonate membrane filter (Isopore, Millipore, MA, USA) pre-coated with Pt for electron charge avoidance was positioned on a suction filtration apparatus, and carbon fibers were collected onto the filter. The morphology of the fibers was determined by SEM examination, and the number of carbon fibers was counted.

### Gross pathological examination and organ weight

For the carcinogenicity evaluation, rats that were not subjected to carbon fiber analysis in the lung were exsanguinated from the abdominal aorta under deep isoflurane anesthesia. All organs and tissues were weighed and then preserved in a 10% buffered formalin solution for gross pathological examination.

### Histopathological examination

For the histopathological examination, the heart, spleen, trachea, lungs (including bronchi), liver, kidney, diaphragm, peritoneum, vertical lymph nodes, and proliferative lesions were sliced into 5-mm-thick sections, embedded in paraffin, and then processed with hematoxylin and eosin (H&E) staining for histopathological examination. The terminology used in this study confirms to the INHAND Project [29] and the following documents [30–33].

### Statistical analysis

For comparisons of the vehicle and treated groups, the homogeneity of variance was analyzed by Bartlett’s test (p < 0.05). If homogeneous, the data were analyzed using the parametric Dunnett’s test (two-sided); if not homogeneous, the data were analyzed by the non-parametric Steel’s test (two-sided). For comparisons of the untreated group vs. the vehicle group and for comparisons between the two groups supplied with the same doses of VGCF™-H and MWNT-7, the means were analyzed using the F-test. If the differences in means were non-significant, a Student's t-test (two-sided) was used; however, if the differences in the means were significant in the F-test, a Welch’s t-test (two-sided) was used. For the histopathological analysis, Fisher's exact test (one-sided) was used to evaluate the frequency of occurrence, and Wilcoxon’s test (two-sided) was used to evaluate the degree. The *P*-values < 0.05 were considered statistically significant.

### Supplementary Information

**Additional file 1**. Supplementary figure and tables.

## Data Availability

All data related to this study are publicly available upon reasonable request to the corresponding author.

## References

[1] Mercer RR, Scabilloni JF, Hubbs AF, Wang L, Battelli LA, McKinney W (2013). Extrapulmonary transport of MWCNT following inhalation exposure. Part Fibre Toxicol.

[2] Rittinghausen S, Hackbarth A, Creutzenberg O, Ernst H, Heinrich U, Leonhardt A (2014). The carcinogenic effect of various multi-walled carbon nanotubes (MWCNTs) after intraperitoneal injection in rats. Part Fibre Toxicol.

[3] Sargent LM, Porter DW, Staska LM, Hubbs AF, Lowry DT, Battelli L (2014). Promotion of lung adenocarcinoma following inhalation exposure to multi-walled carbon nanotubes. Part Fibre Toxicol.

[4] Kasai T, Umeda Y, Ohnishi M, Mine T, Kondo H, Takeuchi T (2015). Lung carcinogenicity of inhaled multi-walled carbon nanotube in rats. Part Fibre Toxicol.

[5] Numano T, Higuchi H, Alexander DB, Alexander WT, Abdelgied M, El-Gazzar AM (2019). MWCNT-7 administered to the lung by intratracheal instillation induces development of pleural mesothelioma in F344 rats. Cancer Sci.

[6] International Agency for Research on Cancer, IARC Working Group on the Evaluation of Carcinogenic Risks to Humans. Some nanomaterials and some fibres [Internet]. 2017 [cited 2021 May 27]. Available from: http://www.ncbi.nlm.nih.gov/books/NBK436610/

[7] Nagai H, Okazaki Y, Chew SH, Misawa N, Yamashita Y, Akatsuka S (2011). Diameter and rigidity of multiwalled carbon nanotubes are critical factors in mesothelial injury and carcinogenesis. Proc Natl Acad Sci.

[8] Sakamoto Y, Nakae D, Fukumori N, Tayama K, Maekawa A, Imai K (2009). Induction of mesothelioma by a single intrascrotal administration of multi-wall carbon nanotube in intact male Fischer 344 rats. J Toxicol Sci.

[9] Takagi A, Hirose A, Futakuchi M, Tsuda H, Kanno J (2012). Dose-dependent mesothelioma induction by intraperitoneal administration of multi-wall carbon nanotubes in p53 heterozygous mice. Cancer Sci.

[10] DeLorme MP, Muro Y, Arai T, Banas DA, Frame SR, Reed KL (2012). Ninety-day inhalation toxicity study with a vapor grown carbon nanofiber in rats. Toxicol Sci.

[11] Kasai T, Umeda Y, Ohnishi M, Kondo H, Takeuchi T, Aiso S (2015). Thirteen-week study of toxicity of fiber-like multi-walled carbon nanotubes with whole-body inhalation exposure in rats. Nanotoxicology.

[12] Numano T, Sugiyama T, Kawabe M, Mera Y, Ogawa R, Nishioka A (2021). Lung toxicity of a vapor-grown carbon fiber in comparison with a multi-walled carbon nanotube in F344 rats. J Toxicol Pathol.

[13] Suzui M, Futakuchi M, Fukamachi K, Numano T, Abdelgied M, Takahashi S (2016). Multiwalled carbon nanotubes intratracheally instilled into the rat lung induce development of pleural malignant mesothelioma and lung tumors. Cancer Sci.

[14] Takanobu K, Aiso S, Umeda Y, Senoh H, Saito M, Katagiri T (2015). Background data of spontaneous tumors in F344/DuCrlCrlj rats. Sangyo Eiseigaku Zasshi.

[15] Hojo M, Maeno A, Sakamoto Y, Ohnuki A, Tada Y, Yamamoto Y (2022). Two-year intermittent exposure of a multiwalled carbon nanotube by intratracheal instillation induces lung tumors and pleural mesotheliomas in F344 rats. Part Fibre Toxicol.

[16] Schinwald A, Donaldson K (2006). Use of back-scatter electron signals to visualise cell/nanowires interactions in vitro and in vivo; frustrated phagocytosis of long fibres in macrophages and compartmentalisation in mesothelial cells in vivo. Part Fibre Toxicol.

[17] Donaldson K, Murphy FA, Duffin R, Poland CA (2010). Asbestos, carbon nanotubes and the pleural mesothelium: a review and the hypothesis regarding the role of long fibre retention in the parietal pleura, inflammation and mesothelioma. Part Fibre Toxicol.

[18] Murphy FA, Poland CA, Duffin R, Al-Jamal KT, Ali-Boucetta H, Nunes A (2011). Length-dependent retention of carbon nanotubes in the pleural space of mice initiates sustained inflammation and progressive fibrosis on the parietal pleura. Am J Pathol.

[19] Fukushima S, Kasai T, Umeda Y, Ohnishi M, Sasaki T, Matsumoto M (2018). Carcinogenicity of multi-walled carbon nanotubes: challenging issue on hazard assessment. J Occup Health.

[20] Anatomy, physiology and function of the nasal cavities in health and disease.pdf. 10.1016/s0169-409x(97)00058-6 10837577

[21] Sabaitis CP, Leong BKJ, Rop DA, Aaron CS (1999). Validation of intratracheal instillation as an alternative for aerosol inhalation toxicity testing. J Appl Toxicol.

[22] Sakamoto Y, Hojo M, Kosugi Y, Watanabe K, Hirose A, Inomata A (2018). Comparative study for carcinogenicity of 7 different multi-wall carbon nanotubes with different physicochemical characteristics by a single intraperitoneal injection in male Fischer 344 rats. J Toxicol Sci.

[23] Nagano K, Nishizawa T, Umeda Y, Kasai T, Noguchi T, Gotoh K (2011). Inhalation carcinogenicity and chronic toxicity of indium-tin oxide in rats and mice. J Occup Health.

[24] Organization for Economic Cooperation and Development (OECD) Section 4 of the OECD guidelines for the testing of chemicals: Carcinogenicity Studies, 2018, guideline 451.

[25] Numano T, Morioka M, Higuchi H, Uda K, Sugiyama T, Hagiwara T (2020). Effects of administering different vehicles via single intratracheal instillation on responses in the lung and pleural cavity of Crl:CD(SD) rats. J Toxicol Pathol.

[26] Iida M, Watanabe K, Tsurufuji M, Takaishi K, Iizuka Y, Tsurufuji S (1992). Level of neutrophil chemotactic factor CINC/gro, a member of the interleukin-8 family, associated with lipopolysaccharide-induced inflammation in rats. Infect Immun.

[27] Handa O, Naito Y, Yoshikawa T (2006). Rat cytokine-induced neutrophil chemoattractant-1 (CINC-1) in inflammation. J Clin Biochem Nutr.

[28] Kohyama N, Suzuki Y (1991). Analysis of asbestos fibers in lung parenchyma, pleural plaques, and mesothelioma tissues of North American insulation workers. Ann N Y Acad Sci.

[29] Renne R, Brix A, Harkema J, Herbert R, Kittel B, Lewis D (2009). Proliferative and nonproliferative lesions of the rat and mouse respiratory tract. Toxicol Pathol.

[30] Boorman GA, Suttie AW, Eustis SL, Elwell MR, Mackenzie WF, Eininger JR, et al. Boorman’s pathology of the rat, 2nd edn (2017).

[31] Boorman GA, Eustis SL, Elwell MR, Montgomery Jr. CA, Mackenzie WF. Pathology of the fischer rat. 1990.

[32] Suttie AW (2006). Histopathology of the spleen. Toxicol Pathol.

[33] Shin-dokusei soshiki byorigaku. 2017.

