# Supplementary material for: Difference in carcinogenicities of two different vapor grown carbon fibers with different physicochemical characteristics induced by intratracheal instillation in rats
Source: Part Fibre Toxicol. 2023 Sep 28;20:37. doi: 10.1186/s12989-023-00547-5 (PMC10537556; doi:10.1186/s12989-023-00547-5)
Supplement: Supplementary file 1 — Additional file 1. Supplementary figure and tables. [file 12989_2023_547_MOESM1_ESM.docx]

**Figure S1 Distribution of lengths and diameters of MWNT-7 and VGCF™-H fiber**

(B)

(A)

(D)

(C)

These histograms indicated the length and diameter of MWNT-7 fibers (A and B), VGCF™-H fibers (C and D).

**Table S1 Iron and the other material contents in VGCF™-H and MWNT-7 fiber**

|  | Element | Mg | Al | Si | P | S | Cl | K | Ca | Ti | V | Cr | Mn | Fe | Co | Ni | Cu | Zn | Mo | W |
| --- | --- | --- | --- | --- | --- | --- | --- | --- | --- | --- | --- | --- | --- | --- | --- | --- | --- | --- | --- | --- |
| VGCF™-H fiber | Measurement Conc. (ppm) | ND | ND | ND | ND | ND | ND | ND | ND | ND | ND | ND | ND | 9.7 | ND | ND | ND | ND | ND | ND |
| MWNT-7 fiber | Measurement Conc. (ppm) | ND | ND | ND | ND | 441 | ND | ND | ND | ND | 44 | ND | ND | 4200 | ND | ND | ND | ND | ND | ND |

Element contents of MWNT-7 and VGCF™-H fibers were determined by X-ray fluorescent analysis

**Table S2 Total surface area of carbon fibers in the lungs of male rats**

| **Test article** | **Total dose** | | **week 13 of the experimental period** | | |  | **week 104 of the experimental period** | | | |
| --- | --- | --- | --- | --- | --- | --- | --- | --- | --- | --- |
|  | **(mg/kg)** | | **(10^-6^ m^2^/g lung)** | | |  |  | **(10^-6^ m^2^/g lung)** | | |
| **Control** | **0** |  |  | **-** |  |  |  |  | **-** |  |
| **MWNT-7** | **0.128** | **(5)** | **387.5** | **±** | **65** |  | **(5)** | **100** | **±** | **22.5** |
| **MWNT-7** | **0.64** | **(5)** | **2407.5** | **±** | **200** |  | **(5)** | **1102.5** | **±** | **215** |
| **MWNT-7** | **3.2** | **(5)** | **11462.5** | **±** | **1015** |  | **(0)** |  | **-** |  |
| **VGCF™-H** | **0.128** | **(5)** | **243** | **±** | **31.5** |  | **(5)** | **94.5** | **±** | **27** |
| **VGCF™-H** | **0.64** | **(5)** | **1521** | **±** | **184.5** |  | **(5)** | **249** | **±** | **60** |
| **VGCF™-H** | **3.2** | **(5)** | **6331.5** | **±** | **450** |  | **(5)** | **1596** | **±** | **1662** |

Number in parentheses indicates the number of animals examined.

The values indicate as mean ± S.D.

**Table S3 Total surface area of carbon fibers in the lungs of female rats**

| **Test article** | **Total dose** |  | **week 13 of the experimental period** | | | | **week 104 of the experimental period** | | | |
| --- | --- | --- | --- | --- | --- | --- | --- | --- | --- | --- |
|  | **(mg/kg)** |  | **(10^-6^ m^2^/g lung)** | | |  |  | **(10^-6^ m^2^/g lung)** | | |
| **Control** | **0** |  |  | **-** |  |  |  |  | **-** |  |
| **MWNT-7** | **0.128** | **(5)** | **225** | **±** | **32.5** |  | **(5)** | **87.5** | **±** | **15** |
| **MWNT-7** | **0.64** | **(5)** | **1465** | **±** | **85** |  | **(5)** | **912.5** | **±** | **120** |
| **MWNT-7** | **3.2** | **(5)** | **7715** | **±** | **1070** |  | **(5)** | **6285** | **±** | **857.5** |
| **VGCF™-H** | **0.128** | **(5)** | **199.5** | **±** | **31.5** |  | **(4)** | **97.5** | **±** | **18** |
| **VGCF™-H** | **0.64** | **(5)** | **972** | **±** | **220.5** |  | **(5)** | **372** | **±** | **48** |
| **VGCF™-H** | **3.2** | **(5)** | **4849.5** | **±** | **396** |  | **(5)** | **505.5** | **±** | **273** |

Number in parentheses indicates the number of animals examined.

The values indicate as mean ± S.D.
